# Advancements in understanding tumor-resident bacteria and their application in cancer therapy

**DOI:** 10.1186/s40779-025-00623-1

**Published:** 2025-07-25

**Authors:** Yi-Chen Luo, Xiu-Ting Huang, Rui Wang, Yu-Jing Lin, Jia-Xin Sun, Ke-Feng Li, De-Yun Wang, Yan Yan, Yong-Kang Qiao

**Affiliations:** 1 Centre for Biological Science and Technology, Key Laboratory of Cell Proliferation and Regulation Biology of Ministry of Education, Department of Biology, Faculty of Arts and Sciences, Guangdong Zhuhai-Macao Joint Biotech Laboratory, Beijing Normal University, Zhuhai, 519087 Guangdong China; 2 Department of Pathology, the Fifth Affiliated Hospital, Sun Yat-Sen University, Zhuhai, 519000 Guangdong China; 3 Guangdong-Hong Kong-Macao University Joint Laboratory of Interventional Medicine, the Fifth Affiliated Hospital, Sun Yat-Sen University, Zhuhai, 519000 Guangdong China; 4 School of Medicine, Sun Yat-Sen University, Shenzhen, 518055 Guangdong China; 5 Faculty of Applied Sciences, Macao Polytechnic University, Macau, SAR 999078 China; 6 Department of Otolaryngology, Yong Loo Lin School of Medicine, National University Health System, National University of Singapore, Singapore, 119228 Singapore

**Keywords:** Intratumoral microbiome, Intracellular bacteria, Cancer progression, Immune modulation, Cancer therapeutics

## Abstract

Recent advances in next-generation sequencing and bioinformatics have driven growing interest in the distinct roles of intratumoral microbiota, particularly intracellular bacteria, during tumor evolution. These bacteria increase the likelihood of metastasis, play important roles in cancer progression, and impact therapy efficiency. The present review explores the sources, mechanisms of invasion into cancer cells, and potential survival strategies of intracellular bacteria in neoplasms, highlighting their critical role in cancer development. We also examine the heterogeneity and intricate interplay of intratumoral microbial communities with immune and cancer cells, emphasizing their potential roles in modulating host genetics, epigenetics, and immunity. Finally, we discuss novel approaches to targeting intracellular bacteria, particularly engineered drug delivery systems, and synthetic biology, which aim to enhance bacterial clearance, reprogram the tumor immune microenvironment, and enhance the efficacy of chemotherapy and immunotherapy. As a result, this review provides new insights to guide future investigations and support the development of microbiota-based interventions in oncology.

## Background

The human microbiota comprises a complex community that includes bacteria, viruses, fungi, and bacteriophages, which colonize throughout the body, with the vast majority found on the external and internal surfaces, while others reside deep within the organs [1]. Although recent estimation has revised the human-to-bacteria cell ratio from the previously proposed 1:10 to 1:1 [2, 3], the microbiota remains extensive and plays a critical role in maintaining health by aiding digestion, modulating immune responses, and contributing to metabolism [4–7]. Over the past decade, advances in sequencing technologies have spurred interest in the role of microorganisms, including not only pathogens but also commensals that were previously considered passive bystanders, in the development of diseases such as cancer [7–10]. The influence of certain microbial populations on tumor progression extends beyond their site of origin, affecting distant tissues via genetic, epigenetic, immune, and metabolic mechanisms [11–13]. Additionally, variation in microbial composition across different cancer stages suggests a symbiotic relationship between the microbiome and malignant cells in multiple cancer types, including colorectal, oral, pancreatic, and breast cancers [14–17], holding potential as biomarkers for diagnosis, prognosis, and therapeutic efficacy [17–22].

Despite notable progress in the field, the precise mechanisms by which microbes influence cancer progression and prognosis remain incompletely defined. Most current research has focused on the gut microbiome, which has been linked to both tumor initiation and progression, as well as treatment responses in cancers originating within and outside the gastrointestinal tract [17, 23]. This effect is largely attributed to the microbiota’s ability to modulate the tumor immune microenvironment (TIME) by influencing the infiltration and activation of immune cells. Consequently, the gut microbiome can alter tumor growth dynamics and therapeutic sensitivity in both beneficial and detrimental ways [24, 25]. One proposed mechanism involves cancer-associated microbes producing immunomodulatory metabolites, such as short-chain fatty acids (SCFAs), which may enhance anti-tumor immunity or, conversely, facilitate cancer progression and immune evasion [26–28].

Compared to the relatively well-characterized relationship between the gut microbiome and cancer development, the functional role of tumor-resident microorganisms in modulating cancer progression and therapeutic outcomes remains poorly understood. The presence of inconsistencies across studies has impeded the establishment of a consensus regarding the pivotal role of tumor-resident microbiota in cancer progression [10, 26, 29–32]. One potential explanation for these discrepancies is the variability in sampling cohorts. Evidence indicates that human microbiota, both inside and outside the gut, can be influenced by environmental factors such as diet, pollutants, and lifestyle exposures, as well as host factors including age, genetics, and disease status [33–36]. These variations across different study cohorts may fundamentally affect what kinds of microorganisms can be detected and how they change with cancer progression. Furthermore, distinct cancer subtypes may present unique microbial signatures, as demonstrated by research on breast cancer, which revealed differing patterns between triple-negative and triple-positive samples [37]. Therefore, when comparing findings across studies, it is essential to consider differences in diagnostic criteria and cancer subtype classifications, as these variations may significantly influence microbiome signatures. Finally, in addition to variations in study cohorts, factors such as sampling protocols, quality control measures, contamination from host tissues, and sequencing depth may also contribute to the inconsistencies observed in the existing literature [38]. Despite the inconsistencies, an increasing number of studies have reported the presence of intratumoral microbiomes and highlighted their association with cancer stages, subtypes, responses to therapy, and patient outcomes [11, 39, 40]. These findings suggest a potential critical role for tumor-resident microorganisms in cancer progression. Notably, recent discoveries have underscored that the majority of intratumoral microbes reside within the cytoplasm of cancer cells, significantly enhancing metastatic potential [10, 12, 29]. This revelation has shifted the focus of research toward the significant role of intracellular bacteria in cancer biology. In this regard, the priority of bacterial invasion into cancer cells and its heterogeneity throughout tumor evolution raises several fundamental questions: 1) the sources of intratumoral microbes; 2) the mechanisms establishing bacterial selective colonization and persistence inside cancer cells; 3) the dynamics of intracellular microbes and cancer cell interactions, along with their consequences for tumor progression and treatment; and 4) the approaches that can be taken to target intracellular microbes and inhibit bacterial-tumor crosstalk. Although there has been progress in this area, the answers to these questions remain elusive.

This review focuses on the complex role of intratumoral microbiota in cancer, with a particular interest in commensals, mutualists, and opportunistic pathogens. The first section summarizes studies reporting the association between intratumoral microbiota and various cancer types, highlighting the heterogeneity of microbial communities and their implications in cancer diagnosis and prognosis. The following sections assess the current knowledge and outline hypotheses regarding the origins and mechanisms underlying the selective colonization of cancer cells by intratumoral microbes. This includes consideration of potential cellular machinery involved, such as endocytosis, cytoskeletal dynamics, and autophagy. The subsequent discussion addresses the mechanisms through which intratumoral microorganisms modulate tumor dynamics, including their roles in shaping TIME and influencing host genetic and epigenetic regulation. Finally, recent advances in research tools and emerging therapeutic strategies targeting the intratumoral microbiome are presented, aiming to provide insights into future research directions and novel anti-cancer interventions.

## Heterogeneity of the intratumoral microbiota: implications for cancer diagnosis and prognosis

The discovery of the intratumoral microbes can be traced back to the early twentieth century when Peyton Rous [41, 42] showed that healthy chickens could develop sarcomas when injected with the filtered cell-free extract from sarcoma tissues, demonstrating that a transmissible agent, later named the Rous sarcoma virus, could induce cancer. This was the first cornerstone for the study of oncogenic microorganisms and led to the discovery of the first proto-oncogene, *c-src*, in the 1970 s [43]. Thereafter, various cancer-associated viruses were identified and recognized as Class I carcinogens for human cancer by the International Agency for Research on Cancer, which includes the high-risk human papillomavirus (HPV), Epstein-Barr virus, hepatitis B and C viruses, human immunodeficiency virus type 1, human T-cell lymphotropic virus, and Kaposi’s sarcoma-associated herpesvirus [44]. In the past few decades, the mechanisms underlying virus-induced carcinogenesis have been extensively studied, including the integration of viral DNA into the host genome, the expression of viral proteins that target tumor suppressors, the activation of oncogenic pathways, and the regulation of immune (both innate and adaptive) and metabolic pathways, all of which lead to genome instability, impaired DNA damage repair, immune evasion, and malignant transformation [45–47].

Besides viruses, *Helicobacter pylori* (*H. pylori*) is the only bacterium recognized as a Class I carcinogen. Although contradictory observations exist, numerous cohort studies support a significant correlation between *H. pylori* infection and the development of gastric cancer [48–50]. It has been shown that the colonization of *H. pylori* in gastric mucosa can induce chronic inflammation, modulate the host immune response, cause DNA damage, and induce epigenetic changes in gastric epithelial cells through its virulence factors [e.g., cytotoxic-associated gene A (CagA), cytotoxic-associated gene Y (CagY), and vacuolating cytotoxin A (VacA)], as well as interfere with several host signaling pathways to regulate cellular proliferation and apoptosis [51–55]. Additionally, *H. pylori* can infect gastric epithelial stem cells and modulate relevant pathways important for the transition from chronic atrophic gastritis to gastric cancer [56, 57].

Strikingly, while pathogens such as high-risk HPV and *H. pylori* are strongly linked to carcinogenesis, only a small fraction of infected individuals eventually develop invasive cancers [48, 58]. This suggests that additional cofactors may contribute to cancer progression, among which the local microbial dysbiosis may play a critical role. For example, in cervical cancer, cervicovaginal microbiota dysbiosis, a departure from a *Lactobacillus*-dominated healthy community, facilitates persistent HPV infection and chronic inflammation, thereby promoting tumorigenesis [59–61]. Similarly, significant differences in microbial composition were observed between gastric cancer and its precancerous stages, with cancer samples more frequently containing intestinal and oral microbes [62, 63]. Re-analyses of large-scale cancer datasets, including the Integration Mutation Profiling of Actionable Cancer Targets (IMPACT) and The Cancer Genome Atlas (TCGA), support these findings by identifying several enriched genera (e.g., *Bacteroides*, *Helicobacter*, *Lactobacillus*, *Prevotella*, and *Streptococcus*) and species (e.g., *H. pylori*, *Staphylococcus cohnii*, *Brachybacterium faecium*, and *Micrococcus luteus*) in gastric tumors versus non-malignant samples [64, 65]. These results collectively point to an association between local microbial alterations and cancer progression, although the underlying mechanisms remain to be fully elucidated.

### Heterogeneity of the intratumoral microbiota

Microorganisms within the tumor microenvironment (TME), similar to those associated with HPV-related cervical cancer and *H. pylori*-related gastric cancer, are increasingly recognized across numerous cancer types for their diagnostic and prognostic importance, regardless of the presence of known carcinogens, as summarized in Table 1 [20–22, 26, 29, 40, 63–107]. Nejman et al. [10] analyzed 1010 tumor samples and 516 healthy tissue samples from 7 cancer types, including breast, lung, ovarian, pancreatic, melanoma, bone, and brain tumors, and reported bacterial enrichment in all tumor types compared with both techniques and paraffin controls. This finding indicates that microorganisms are present not only in tumors in externally exposed anatomical sites but also in those that are not, such as ovarian cancer (OV), glioblastoma multiform, and bone cancer. Using 16S rRNA sequencing, the authors demonstrated that intratumoral microbial compositions are cancer-type specific, with significant differences in beta diversity and distinct microbiome profiles at both the order and species levels [10]. These variations likely reflect the diverse origins of intratumoral microorganisms and may be influenced by the unique microenvironments of each cancer type. For example, certain intratumoral bacteria are closely linked to tumor-specific metabolic features, such as hydroxyproline degradation in bone tumors and the processing of chemicals from cigarette smoke in lung cancer [10]. These metabolic capabilities may promote the preferential colonization of specific bacterial taxa in certain cancer types, offering a possible explanation for the heterogeneity of the cancer-specific intratumoral microbiota. Whether these microbial-associated metabolic pathways are involved in cancer progression needs to be validated.

**Table 1:** Intratumoral microbiota identified in different cancer types

Cancer type	Sequencing methods	Clinical samples	Intratumoral microbiota identified	Clinical significance (diagnostic/prognostic potentials)	Mechanisms	Reference
Anal squamous cell carcinoma (ASCC)	Real-time qPCR for *Fusobacterium nucleatum* 16 rRNA	Surgical samples of abdominoperineal resection from ASCC patients	*Fusobacterium nucleatum* was detected with high abundance in 33.1% of ASCC samples	High *Fusobacterium nucleatum* load was identified as an independent favorable prognostic factor for ASCC	–	[66]
Breast cancer	16S rRNA sequencing	Breast cancer samples	*Fusobacterium nucleatum* was detected in 30% of analyzed breast tumors and was predominately found in samples with high Gal–GalNAc expression levels	–	*Fusobacterium nucleatum* may colonize breast tumors via Fap2–Gal–GalNAc binding, thereby suppressing tumor-infiltrating T cells, as well as promoting tumor growth and metastasis	[67]
	16S rRNA sequencing	Tissue specimens from breast cancer tumors, tumor-adjacent normal, high-risk, and healthy controls	The tumor tissues were enriched with *Pseudomonas*, *Proteus*, *Porphyromonas*, and *Azomonas*, while the tumor-adjacent normal tissues were enriched with *Propionibacterium* and *Staphylococcus*. The healthy control tissue was further marked with the presence of *Stenotrophomonas* and *Caulobacter*	Multiple bacterial genera, such as *Porphyromonas*, *Lacibacter*, *Ezakiella*, *Fusobacterium*, and *Stenotrophomonas* were significantly associated with prognostic breast tumor features, including tumor stage, subtypes, receptor expression status, and metastatic potential	The depletion of *Propionibacterium* and *Staphylococcus* may promote a tumor-supportive environment, while the reduction of *Streptococcus* might be linked to deficient T-cell activation signals	[26]
	16S rRNA gene sequencing data extracted from the database	Benign breast tumors, malignant breast cancer, nipple aspirate fluid of breast cancer survivors, and healthy controls	*Bacteroides fragilis* was consistently detected in all the breast tissue samples	–	Enterotoxigenic *Bacteroides fragilis* may have oncogenic effects on breast cancer, through the regulation of β-catenin and Notch1 pathways	[68]
	16S rRNA sequencing	Breast cancer tissues with paired adjacent normal breast tissues and lymph node metastasis	The breast cancer tissue contained a significantly higher abundance of *Enterococcus* and *Streptococcus*, and closely clustered with lymph node metastasis	–	Intracellular bacteria residing in breast cancer cells facilitate metastasis by promoting the survival of circulating cancer cells through the regulation of the actin cytoskeleton	[29]
Cholangiocarcinoma (CCA)	16S rRNA sequencing	Freshly frozen tissues collected from CCA patients	Gammaproteobacteria were significantly higher in both gemcitabine- and cisplatin-resistance groups compared to sensitive groups	–	CCA intratumoral microbiome correlated with metabolic profiles, which may affect chemotherapeutic sensitivity	[69]
	16S rRNA sequencing, single-cell RNA sequencing (scRNA-seq)	Surgical samples from intrahepatic cholangiocarcinoma (ICC) tumor and precancerous tissues	*Paraburkholderia fungorum* was significantly higher in the paracancerous tissues DNAs for *Klebsiella pneumoniae*, *Pseudomonas azotoformans*, *Staphylococcus capitis*, and *Paraburkholderia fungorum* were present in ICC tissues	–	*Paraburkholderia fungorum* may inhibit tumor growth through alanine, aspartate, and glutamate metabolism	[70]
Colorectal cancer (CRC)	16S rRNA sequencing	Fresh-frozen primary CRC tumors and paired liver metastasis	The same *Fusobacterium* species, along with other primary cancer microbes including *Bacteroides fragilis*, *Bacteroides thetaiotaomicron*, *Prevotella intermedia*, and *Selenomonas sputigena* persisted in the liver metastases	–	*Fusobacterium* may contribute to tumor progress, as evidenced by a murine xenograft model treated with antibiotics	[71]
	16S rRNA sequencing	Tissue biopsies from patients with CRC or adenoma, as well as adjacent normal tissues	*Peptostreptococcus* and *Fusobacterium* were enriched in CRC compared with CRC-adjacent normal tissues Significant variations in microbial communities across different biopsies from the same neoplasia were identified. The variation in abundance of *Prevotella* and *Fusobacterium* decreased along the adenoma-carcinoma sequence, whereas *Parvimonas* and *Bacteroides* variation reversed along the sequence *Bacteroides*, *Peptostreptococcus*, and *Clostridium* were associated with *KRAS* mutation *Gallionella* and *Dechloromonas* were associated with microsatellite instability (MSI)	The presence of specific bacteria, like *Fusobacterium nucleatum*, was associated with both hereditary and sporadic MMRd subtypes	The heterogeneity of the microbial community within a single tumor or adenoma might affect CRC progression and could be linked to genetic mutations like *KRAS* and MSI	[72]
	Whole-transcriptome RNA sequencing with rRNA depletion	CRC tumor biopsies	Bacteriodota*,* Firmicutes*, and* Fusobacteriota were the common phyla in CRC tumors. Oral taxa including *Fusobacterium* species, *Gemella morbillorum*, *Parvimonas micra*, and *Peptostreptococcus stomatis* were prevalent in CRC tumors. Oral bacteria were enriched among right-sided, microsatellite-unstable, and *BRAF*-mutant tumors	–	The prevalence of *Fusobacterium animalis* was associated with collagen- and immune-related pathways in the mesenchymal CRC subtype, suggesting a possible role of *Fusobacterium animalis* in cancer progression	[73]
	16S rRNA sequencing	Tumor and paired adjacent non-malignant fresh frozen tissue specimens prospectively collected from yoCRC (age < 50 years) and aoCRC (age > 60 years) patients	yoCRC tumors were enriched with *Akkermansia* and *Bacteroides*, whereas aoCRC tumors were enriched with *Bacillus, Staphylococcus*, *Listeria*, *Enterococcus*, *Pseudomonas*, *Fusobacterium*, and *Escherichia/Shigella*	Abundance of intratumoral *Fusobacterium* and *Akkermansia* correlated with overall survival in yoCRC	–	[22]
	Targeted PCR	Tumor-derived DNA samples from the Australasian CRC Family Registry, Melbourne Collaborative Cohort Study, and the Applying Novel Genomic approaches to Early-onset and suspected Lynch Syndrome colorectal and endometrial cancers study	*pks*^+^ *Escherichia coli* (*E. coli*), Enterotoxigenic *Bacteroides fragilis*, and *Fusobacterium nucleatum* were associated with clinicopathological and molecular features of CRC	–	Colibactin-producing *E. coli* exposures may be related to DNA damage leading to APC: c.835–8 A > G somatic mutation DNA MMRd in CRC could be important for the intratumoral colonization of *Fusobacterium nucleatum*	[21]
Cervical cancer	mRNA sequencing data and microbiome data extracted from the TCGA CESC cohort	Primary tumor samples from the TCGA-CESC cohort	*Frigoribacterium*, *Robiginitomaculum*, *Actinobaculum*, *Microbispora*, *Klebsiella*, *Micromonospora*, and *Hylemonella* were increased in metastasis vs. non-metastasis group, whereas *Leeia*, *Acetonema, Kobuvirus*, *Steroidobacter, Methylobacter*, *Tobamovirus*, *Tymovirus*, and *Marinomonas* were increased in non-metastasis vs. metastasis group	Intratumoral microbiome characterized by *Methylobacter*, *Robiginitomaculum*, *Klebsiella*, *Micromonospora*, and *Microbispora* predicted a poor prognosis, whereas *Mythylobacter* predicted a better prognosis for cervical cancer	–	[74]
Esophageal cancer	IHC and real-time qPCR for *Porphyromonas gingivalis*	Surgical cancerous and adjacent tissue samples from ESCC patients	*Porphyromonas gingivalis* was detected in 71% of ESCC tissues, compared with that in 12% of adjacent tissues	Intratumoral *Porphyromonas gingivalis* levels were correlated with tumor differentiation status, metastasis, and overall survival rate	–	[75]
	qPCR for *Fusobacterium nucleatum*	Formalin-fixed paraffin-embedded esophageal cancer specimens collected from patients with ESCC (92%), EAC (3.7%), and others (4.0%)	*Fusobacterium nucleatum* DNA was detected in 23% of esophageal tumors and was significantly higher than matched normal esophageal mucosa	Intratumoral *Fusobacterium nucleatum* levels were associated with shorter survival, serving to be a potential prognostic biomarker	Expression of the chemokine CCL20 was significantly higher in *Fusobacterium nucleatum*-positive tumors, which might indicate a possible role of *Fusobacterium nucleatum* in cancer progression through the activation of chemokines	[76]
	16S rRNA sequencing	Esophageal samples from patients, consisting of normal squamous controls, non-dysplastic, dysplastic Barrett’s oesophagus, and EAC	*Lactobacillus fermentum* was enriched in EAC compared with Barrett’s esophagus samples and control samples	–	–	[77]
	qPCR for *Fusobacterium nucleatum*	Fresh frozen or FFPE tumor tissues collected from ESCC patients in two independent cohorts	*Fusobacterium nucleatum* levels were significantly higher in cancer tissues compared to the adjacent normal tissues *Fusobacterium nucleatum* was more abundant in ESCC tumors at advanced stages vs. those at earlier stages	High levels of *Fusobacterium nucleatum* had a prognostic significance for predicting poor recurrence-free survival in ESCC patients	*Fusobacterium nucleatum* may contribute to chemoresistance in ESCC patients	[40]
	Real-time qPCR for *Fusobacterium nucleatum*	Clinical specimens from three cohorts including patients who underwent surgical resection before or after chemotherapy	Intratumoral *Fusobacterium nucleatum* levels were significantly higher in chemotherapy non-responders	–	*Fusobacterium nucleatum* invades ESCC cells and promotes autophagy to confer chemoresistance	[78]
	16S rRNA sequencing	Surgically resected tissues from ESCC patients, including patients with STS vs. LTS	At the phylum level, *Actinobacteriota*, *Chloroflexi*, and unidentified Bacteria were significantly higher in STSs vs. LTSs, whereas Fusobacteriota were higher in LTSs. At the genus level, *Lactobacillus*, *Escherichia/Shigella*, *Enterococcus*, *Ralstonia*, and *Syntrophotalea* were significantly higher in STSs, whereas *Leptotrichia* was significantly higher in LTSs	A higher abundance of *Lactobacillus* was independently associated with poor survival	Intratumoral *Lactobacillus* levels positively correlated with PD-L1^+^ epithelial cells and PD-L1^+^ tumor-associated macrophages, suggesting a potential role of *Lactobacillus* in promoting an immunosuppressive microenvironment	[79]
Gastric cancer	16S rRNA sequencing	Gastric mucosal samples from SG, AG, IM, and GC	*Parvimonas micra*, *Dialister pneumosintes*, *Slackia exigua*, *Peptostreptococcus stomatis*, *Prevotella intermedia*, *Fusobacterium nucleatum*, *Prevotella oris,* and *Catonella morbi* were enriched in GC samples compared with precancerous stages	*Peptostreptococcus stomatis*, *Streptococcus anginosus*, *Parvimonas micra*, *Slackia exigua,* and *Dialister pneumosintes* had diagnostic potential to distinguish GC from SG	–	[63]
	Whole exome sequencing (WES)	Tumor vs. paired tumor-adjacent samples from Integration Mutation Profiling of Actionable Cancer Targets (IMPACT) and TCGA cohorts	*Bacteroides*, *Helicobacter*, *Lactobacillus*, *Prevotella*, and *Streptococcus* were enriched in GC samples when compared to non-malignant samples	*Lactobacillus*, *Streptococcus*, *Prevotella*, *Fusobacterium*, *Selenomonas*, and *Porphyromonas* showed enrichment in the MSI-high GC subtype, suggesting a diagnostic potential for cancer subtypes	–	[64]
	RNA sequencing extracted from TCGA STAD project	STAD cancerous and adjacent normal samples from the TCGA database	*Helicobacter pylori*, *Staphylococcus cohnii*, *Brachybacterium faecium*, human mastadenovirus C (HAdV-C), and *Micrococcus luteus* were enriched in STAD tumors. *Kytococcus sedentarius*, *Brachybacterium avium*, *Dolosigranulum pigrum*, and *Staphylococcus cohnii* showed differential abundances between cancer groups with or without metastasis	*Helicobacter pylori*, *Staphylococcus cohnii*, *Brachybacterium faecium*, HAdV-C, and *Micrococcus luteus* had diagnostic ability for STAD. *Kytococcus sedentarius*, *Brachybacterium avium, Dolosigranulum pigrum*, and *Staphylococcus cohnii* were potential prognostic factors for STAD	STAD intratumoral taxa, such as *Staphylococcus saccharolyticus*, *Kytococcus sedentarius*, *Actinomyces oris*, and *Streptococcus sanguinis* may promote metastasis and cancer cell proliferation by affecting host methylation features or regulating host gene expression	[65]
Head and neck squamous cell carcinoma (HNSCC)	16S rRNA sequencing	Matched HNSCC tumor and non-tumor tissues	*Actinomyces* was significantly depleted, while *Parvimonas* was increased in tumor tissues relative to normal tissues	–	*–*	[80]
	RNA sequencing data and intratumoral microbiome data were extracted from the database	Primary tumors vs. solid normal tissues for HNSCC from The Cancer Microbiome Atlas (TCMA) and TCGA	In HNSCC tumors, the levels of *Actinomyces* and *Rothia* were decreased, whereas the level of *Fusobacterium* was increased compared to normal tissues	High levels of intratumoral *Leptotrichia* correlated with an improved prognosis in HNSCC patients	–	[81]
Hepatocellular carcinoma (HCC)	PCR for *Helicobacter*	Liver specimens were collected from control patients, patients with chronic active hepatitis C virus (HCV) without cirrhosis, patients with terminal-stage HCV cirrhosis without HCC, and patients with HCV-positive cirrhosis and HCC	*Helicobacter* 16S rDNA was positive in 90.5% of HCC samples, significantly higher than the other groups	There was an association between *Helicobacter* in the liver and HCV cirrhosis, with or without HCC	–	[82]
	16S rRNA sequencing	Surgical samples from HCC vs. peritumoral vs. normal liver	Proteobacteria, Firmicutes, and Actinobacteriota phyla were enriched, whereas Patescibacteria and Acidobacteriota phyla were decreased in HCC and peritumoral tissues, compared with normal samples. At the class level, *Bacilli* and *Actinobacteria* were increased, whereas *Parcubacteria* and *Acidobacteriae* were decreased in HCC and peritumoral tissues. Gammaproteobacteria was especially abundant in HCC tissues compared to normal controls. Streptococcaceae and *Lactococcus* were enriched in HCC with cirrhosis compared with HCC without cirrhosis. *Streptococcus* was enriched in HBV-positive HCCs, whereas *Staphylococcus* and *Caulobacter* were enriched in HBV-negative HCCs	The random forest prediction model using all microbial features at the class level achieved high predictive power for HCC, suggesting the diagnostic potential of HCC intratumoral microbiota	–	[83]
	16S rRNA sequencing	Tissue samples from HCC and paracancerous tissues	Enterobacteriaceae, *Fusobacterium*, and *Neisseria* were significantly increased in HCC tissues, while the abundances of certain antitumour bacteria such as *Pseudomonas* were decreased	-	Fatty acid and lipid synthesis were significantly enhanced in HCC microbiota, which could affect cancer progression	[84]
	Real-time qPCR for Mycoplasma 16S rDNA	HCC specimens	Mycoplasma DNA was enriched in HCC samples *Mycoplasma hyorhinis* was detected in almost all the HCC samples	*Mycoplasma hyorhinis* infection was associated with poor prognosis of HCC patients	*Mycoplasma hyorhinis* may retrogradely infect the liver via the hepatopancreatic ampulla, invade host cells, and promote HCC initiation and progression by enhancing nuclear ploidy as a result of mitochondrial fission	[85]
	16S rRNA sequencing, droplet digital PCR (ddPCR)	FFPE sections from viral HCC (hepatitis B virus, HBV- or HCV- related) vs. non-viral HCC	Bacteroidales, *Parabacteroides*, *Peptoniphilus*, *Ruminococcus 2*, *Lachnoclostridium*, *Cutibacterium*, [Eubacterium] coprostanoligenes group, and Burkholderiaceae were enriched in viral HCC samples as compared with other groups. *Dolosigranulum*, *Prevotella 9*, *Cutibacterium*, and *Nocardioides* were enriched in HBV-HCC while *Chryseobacterium* was enriched in non-viral HCC as compared with viral HCC. *Cutibacterium* was considered as the representative taxa biomarker in HBV_HCC	–	Increased intratumoral microbiota burden was positively associated with increased tumor-infiltrating CD8^+^ T lymphocyte and myeloid‐derived suppressor cells in viral HCC, suggesting an inhibitory role of intratumoral microbiota in antitumor immunity	[86]
Renal cell carcinoma (RCC)	16S rRNA sequencing	FFPE samples collected from RCC patients, including cancer and paracancerous tissues	*Cyanophora paradoxa*, *Spirosoma navajo*, *Phaeocystis antarctica*, *Euglena mutabilis*, and *Mycoplasma vulturii* were found only in cancer tissues. *Microbacterium*, *Pelomonas*, *Staphylococcus*, *Strepotococcus*, *Leuconostoc garlicum*, *Corynebacterium vitaeruminis*, *Anaerococcus nagyae*, *Ethanoligenens harbinense*, *Neisseria bacilliformis*, *Thermicanus aegyptius*, and *L. mesenteroides* were found only in healthy tissues. *Aeromonas salmonicida*, *Pseudoalteromonas haloplanktis*, *Parageobacillus toebii*, *Trachelomonas volvocinopsis*, *M. mycoides*, and *Halomicrobium mukohataei* were found in all tissue types, but more frequently in cancer tissues	–	–	[87]
	RNA sequencing	Fresh frozen samples collected from normal adjacent renal parenchyma, RCC tumors, and thrombus	RCC tumor tissues were enriched with *Micrococcus luteus*, *Fusobacterium nucleatum*, *Streptococcus agalactiae*, and *Corynebacterium diphtheriae*, compared to adjacent kidney and tumor thrombus	–	The presence of oral microbiome aggregates (especially *Fusobacterium nucleatum*) in tumors was associated with significantly higher PD-L1 expression in the tumor thrombus, suggesting an association of intratumoral oral microbiome and a suppressive TIME	[88]
	16S rRNA sequencing	Frozen samples collected from RCC patients, with paired cancer and adjacent normal tissues	RCC tissues were enriched with *Deinococcus*, *Phyllobacterium*, *Actinomyces*, and *Gordonia*, while adjacent normal tissues were enriched with *Klebsiella*, *Chloroplast*, *Streptophyta*, and *Bifidobacterium*	Decreased relative abundance of *Klebsiella* (AUC = 0.86), *Chloroplast* (AUC = 0.91), and *Streptophyta* (AUC = 0.89) showed high ability to differentiate RCC tumors from normal tissues, and *Chloroplast* showed highest sensitivity of 91.67% and specificity of 83.33%	–	[89]
	16S rRNA sequencing, and real-time PCR	FFPE blocks collected from RCC patients with clear cell (ccRCC), papillary (papRCC), or chromophobe subtypes, and conditionally normal kidney tissues from non-RCC patients	*Tenericutes* phylum was only present in ccRCC and papRCC tumors. *Gemmatimonadetes*, *Chloroflexi*, *Fusobacteria*, *Parcubacteria*, and *Verrucomicrobia* phyla were found only in normal kidney tissues. Gram-negative bacteria were dominant in ccRCC	The high bacterial burden with PU.1⁺ macrophages and CD66b⁺ neutrophils correlated with poor prognosis	Tumors with a high content of PU.1^+^ macrophages and CD66b^+^ neutrophils in the stroma were characterized by a lower bacterial burden, suggesting an association of intratumoral microbiome with TIME	[90]
Lung cancer	16S rRNA sequencing	Bronchoscopy samples collected from the cancerous site, paired contralateral noncancerous site, and healthy controls	*Streptococcus* was significantly more abundant in cancerous samples compared with other samples. *Streptococcus* and *Neisseria* increased from normal to paired noncancerous to cancerous group, whereas *Staphylococcus* and *Dialister* displayed a reverse trend	*Streptococcus* exhibited a moderate prediction potential for lung cancer	–	[91]
	16S rRNA gene sequencing	Bronchoscopy samples collected from lung cancer patients, patients with a benign pulmonary nodule, and healthy controls	Oral taxa such as *Streptococcus*, *Prevotella*, *Veillonella*, and *Rothia* were enriched in the cancerous samples	*Prevotella, Veillonella,* and *Streptococcus* were identified to predict lung cancer, suggesting a diagnostic potential	Oral commensals such as *Veillonella* were associated with cancer-relevant pathways	[92]
	16S rRNA sequencing, RNA sequencing data from TCGA lung cancer cases	Lung tissues from tumor (SCC vs. AD), non-tumor adjacent, or normal lung	Lung cancer microbiota was dominated by Proteobacteria. *Acidovorax*, *Brevundimonas*, *Comamonas*, *Tepidimonas*, *Rhodoferax*, *Klebsiella*, *Leptothrix*, *Polaromonas*, and *Anaerococcus* were differentially regulated in SCC vs. AD tumors. *Acidovorax* was more abundant in smokers and was further enriched in SCC tumors with *TP53* mutations compared to AD tumors	*–*	–	[93]
	16S rRNA sequencing	Surgical samples from patients with pulmonary (SCC or AD) vs. adjacent healthy tissues	Enteric bacteria, potential pathogens, or inflammatory bacteria, such as *Escherichia/Shigella*, *Faecalibacterium*, *Pseudomonas*, unclassified Enterobacteriaceae*, Alloprevotella*, and *Brevundimonas*, were only present in cancerous tissue	–	–	[94]
	16S rRNA gene sequencing	Bronchoscopy samples from lung cancer patients, of whom 89% had a diagnosis of non-small cell lung cancer	Oral commensals such as *Haemophilus*, *Fusobacterium*, *Gemella*, *Prevotella*, and *Granulicatella* were enriched in stage IIIB-IV lung cancer. *Veillonella*, *Prevotella*, and *Streptococcus* were enriched in patients with worse prognosis	*Prevotella, Streptococcus*, *Lactobacillus*, and *Gemella* were associated with poor overall survival (independent of TNM staging)	The lower airway dysbiotic signature was associated with upregulation of the IL-17 inflammatory pathway and other pathways (PI3K, MAPK, ERK), linking to cancer progression	[95]
Nasopharyngeal carcinoma (NPC)	16S rRNA sequencing	Pretreatment tumor biopsy samples were collected from NPC patients, among whom paired patients were compared with or without relapse	*Corynebacterium* and *Staphylococcus* predominated in NPC tumor tissues. Tumors from patients with relapse exhibited a significant increase in *Prevotella* and *Porphyromonas* levels	A high intratumoral bacterial load was associated with poor prognosis in NPC, serving to be a robust prognostic biomarker	A higher intratumoral bacterial load was negatively associated with T-lymphocyte infiltration, suggesting a possible role of NPC intratumoral microbiota in tumor immunity	[96]
Oral cancer	Real-time qPCR for *Fusobacterium nucleatum* 16S rRNA	Oral squamous cell carcinoma (OSCC) tissue samples from two independent cohorts	*Fusobacterium nucleatum* was detected positive in 71.3% and 84.4% of two cohorts, respectively	*Fusobacterium nucleatum* positivity was associated with a favorable prognosis in OSCC patients	*Fusobacterium nucleatum* might be linked to antitumor immunity	[97]
	16S rRNA sequencing	Multiple types of specimens, including saliva, swabs from the surface of tumor tissues, adjacent normal tissues, tumor outer tissues, tumor inner tissues, and lymph nodes, were collected from OSCC patients	*Fusobacterium* was enriched in the outer tumor tissues as compared with normal adjacent tissues. When the outer and inner tumors were compared, *Fusobacterium*, *Neisseria*, *Porphyromonas*, and *Alloprevotella* were more abundant in the outer tumor tissues, while *Prevotella*, *Selenomonas*, and *Parvimonas* were overabundant in the inner tumor tissue. As for the outer tumor microbiome, *Gemella* and *Bacillales* were enriched in T_1_/T_2_-stage patients and the non-lymphatic metastasis group, while *Spirochaetae* and *Flavobacteriia* were enriched in the extranodal extension negative group	Some taxa were associated with clinical stages, suggesting a potential for diagnosis and prognosis	–	[98]
	16S rRNA sequencing	Tissue specimens collected from precancer, early cancer, late cancer, and adjacent tumor tissues	The bacterial composition varied significantly between the precancer, early cancer, and late cancer stages. The cancer group showed an enrichment of the genera *Capnocytophaga*, *Fusobacterium*, and *Treponema*, while the precancer group showed an enrichment of the genera *Streptococcus* and *Rothia*. *Capnocytophaga* was significantly associated with late cancer stages, while *Fusobacterium* was associated with early stages of cancer	*Capnocytophaga* and *Streptococcus* had good diagnostic potentials for OSCC	OSCC-associated microbiota was associated with tumor infiltration of immune cells, indicating a possible role of OSCC microbiome in modulating tumor immunity	[99]
Ovarian cancer (OV)	RNA sequencing data extracted from the TCGA-OV cohort	TCGA-OV cohort	The immune-deficient subtype (clust1) was enriched with 58 microbial species, mainly from *Pseudomonas*, whereas the immune-enriched subtype (clust2) was featured by 11 species. *Achromobacter deleyi, Microcella alkaliphila*, *Devosia sp*. strain LEGU1, *Ancylobacter pratisalsi*, and *Acinetobacter seifertii* showed strong association with M1 macrophages	A prognostic model incorporating 32 microbial signatures was developed using the Cox proportional-hazard model, demonstrating significant prognostic value for patients with OV	The intratumoral microbiota in OV may influence tumor immunity, as demonstrated by the association of microbial composition with immune features, as well as the inhibitory effect of *Acinetobacter seifertii* on macrophage migration in vitro	[100]
	Pan-pathogen array (PathoChip) combined with capture-next generation sequencing	Paired tumor and non-tumor tissues	*Fusobacterium*, *Mycoplasma*, *Chlamydia*, and *Propionibacterium* were detected to be enriched in tumor tissues. OV tumors exhibited significant viral signatures including human papillomavirus and polyomaviruses	–	–	[101]
Pancreatic cancer	16S rRNA sequencing	Pancreatic ductal adenocarcinoma (PDAC) tumors that were positive for bacterial DNA	Gammaproteobacteria*,* mainly Enterobacteriaceae and Pseudomonadaceae families were enriched in human PDAC tumors	–	Gammaproteobacteria can metabolize the chemotherapeutic drug gemcitabine (2’,2’-difluorodeoxycytidine) into its inactive form, 2’,2’-difluorodeoxyuridine, via the expression of a long isoform of the bacterial enzyme cytidine deaminase (CDD_L_). This metabolic activity potentially modulates tumor sensitivity to chemotherapy	[20]
	16S rRNA sequencing	Surgical PDAC tumors	Proteobacteria, Bacteroidetes, and Firmicutes were most abundant and were prevalent in PDAC tumors, of which Genera *Pseudomonas* and *Elizabethkingia* were highly abundant	–	The mouse model studies suggested that the PDAC microbiome could promote tumorigenesis by inducing immune suppression through selective Toll-like receptor activation, leading to T-cell anergy and creating a tolerogenic immune environment	[102]
	16S rRNA sequencing	Archived FFPE tumor specimens obtained from PDAC patients with STS vs. LTS	At the class level, LTS tumors were enriched in Alphaproteobacteria, Sphingobacteria, and Flavobacteria, whereas STS tumors were dominated by Clostridia and Bacteroidea At the genus level, LTS tumors exhibited higher abundances of *Pseudoxanthomonas*, *Saccharopolyspora*, and *Streptomyces* compared to STS tumors At the species level, *Bacillus clausii* and *Saccharopolyspora rectivirgula* were more abundant in LTS tumors than in STS tumors	The intratumoral microbiome signature (*Pseudoxanthomonas-Streptomyces-Saccharopolyspora-Bacillus clausii*) predicted a long-term survivorship in PDAC	The intratumoral microbiota of PDAC engages in cross-talk with the gut microbiome, influencing tumor immune infiltration and ultimately impacting PDAC survival	[103]
	18S rRNA sequencing	Surgical samples from patients with PDAC or pancreatic endocrine tumors (benign disease) vs. healthy pancreatic tissues	*Malassezia* spp. was markedly enriched in PDAC tumors	–	The pathogenic fungi could promote PDAC by driving the complement cascade through the activation of ligation of mannose-binding lectin	[104]
	16S rRNA sequencing	Non-surgical fresh-frozen endoscopic ultrasound (EUS) specimens collected from intervention-naïve pancreatic exocrine tumors	Proteobacteria, Bacteroidetes, Firmicutes, Actinobacteria, and Gammaproteobacteria were identified at the phyla or class levels. At the genus level, *Paracoccus*, *Brevundimonas*, *Prevotella*, *Cutibacterium*, *Streptococcus*, *Fusobacterium*, and *Bifidobacterium* were identified, respectively	The microbial phenotyping via EUS at the time of diagnosis could be utilized to identify intratumoral microbiota signatures for prognosis	–	[105]
Soft tissue sarcomas	Metagenomic sequencing	Tumor and stool samples collected from non-metastatic soft tissue sarcomas patients	Proteobacteria, Bacteroidetes, and Firmicutes were detected in all tumors	The intratumoral viral microbiome correlated with NK cell infiltration and overall survival	–	[106]
Vulvar squamous cell carcinoma (VSCC)	16S rRNA sequencing and qPCR for target bacterial species	Snap-frozen tumor tissue samples collected from VSCC patients	Tumor-promoting bacteria, such as *Fusobacterium nucleatum* and *Pseudomonas aeruginosa* were identified in VSCC tissues	*Fusobacterium nucleatum* and *Pseudomonas aeruginosa* were associated with shorter time to progress in VSCC patients	Neutrophilic inflammation may be permissive for tumor-promoting bacteria growth	[107]

*MSI-high* microsatellite instability-high, *PI3K* phosphoinositide 3-kinase, *MAPK* mitogen-activated protein kinase, *ERK* extracellular signal-regulated kinase, * TNM* tumor-node-metastasis, *AUC* area under the curve, *HBV* hepatitis B virus, *IL* interleukin*, KRAS* Kirsten rat sarcoma viral oncogene homolog, *yoCRC* young-onset colorectal cancer, *aoCRC* average-onset colorectal cancer, *qPCR* quantitative polymerase chain reaction, *16S rRNA* 16S ribosomal RNA, *Gal-GalNAc* D-galactose-β(1–3)-N-acetyl-D-galactosamine, *Fap2 Fusobacterium* adhesin protein 2, *ETBF* enterotoxigenic Bacteroides fragilis, *Notch1* neurogenic locus notch homolog protein 1, *MMRd* mismatch repair deficient, *PD-L1* programmed death-ligand 1, *CESC* cervical squamous cell carcinoma and endocervical adenocarcinoma, *ESCC* esophageal squamous cell carcinoma, *EAC* esophageal adenocarcinoma, *LTS* long-term survival, *STS* short-term survival, *SG* superficial gastritis, *AG* atrophic gastritis, *IM* intestinal metaplasia, *GC* gastric cancer, *STAD* stomach adenocarcinoma, *CHAdv-C* chimpanzee adenovirus C*, FFPE* formalin-fixed paraffin-embedded, *ddPCR* droplet digital PCR, *ccRCC* clear cell renal cell carcinoma, *papRCC* papillary renal cell carcinoma, *SCC* squamous cell carcinoma, *AD* adenocarcinoma, *NK* natural killer, *TIME* tumor immune microenvironment, *TME* tumor microenvironment, *APC* antigen-presenting cell, *IHC* immunohistochemistry, *CCL20* C–C motif chemokine ligand 20

The intratumoral microbial composition also evolves during cancer progression, varying by cancer stage and subtype. Supporting this, Nejman et al. [10] reported significant differences in microbial taxa abundance across breast cancer subtypes, particularly concerning receptor status. Expanding on this finding, Tzeng et al. [26] further demonstrated microbial shifts across breast tissue types (tumor, adjacent healthy, high-risk, and healthy tissues), with varying cancer stages, histological subtypes, and receptor statuses, and reported that microbial genera, such as *Anaerococcus*, *Caulobacter*, and *Streptococcus*, which were central to microbial networks in benign tissues, were often absent in cancer-associated tissues. Notably, bacterial taxa such as *Propionibacterium* and *Staphylococcus* were depleted in tumors, and the abundances of these taxa were negatively correlated with oncogenic immune features, underscoring the complex interplay between the microbiome and tumor immune responses [26]. In colorectal cancer (CRC), the variation in microorganisms, particularly *Prevotella* and *Fusobacterium*, along the adenoma-carcinoma sequence reinforces the idea that the intratumoral microbiota is not static but dynamically evolves with cancer development [72]. These findings shed light on a potential link between microbial changes and cancer progression. Meanwhile, the variability of these microbes complicates our understanding of their roles, raising the hypothesis that specific microbes may selectively contribute to distinct stages of cancer progression, which warrants further investigation.

In addition to complexity, demographic factors such as age, diet, and smoking or drinking behaviors further contribute to the heterogeneity of the intratumoral microbiota among individuals. For example, in CRC, the intratumoral microbial composition varied significantly between young-onset and average-onset CRC patients, with notable differences in microbial genera such as *Akkermansia*, *Bacteroides*, *Staphylococcus*, *Listeria*, *Enterococcus*, *Pseudomonas*, *Fusobacterium*, and *Escherichia/Shigella*, underscoring the significant impact of age on intratumoral microbiome heterogeneity [22]. Similarly, for esophageal adenocarcinoma (EAC), a prospective study analyzing esophageal brushing samples identified 3 microbiome clusters, referred to as “esotypes”. These clusters were primarily defined by differences in the dominant bacterial genera, *Streptococcus* and *Prevotella*, as well as by distinct metabolic profiles involving SCFA metabolism and lipopolysaccharide biosynthesis [108]. In the early stages of EAC, distinct oral-associated taxa were enriched within each ecotype, without inducing global shifts in the overall microbial composition [108], further emphasizing the individual variability in the intratumoral microbiota.

Compellingly, the heterogeneity of the intratumoral microbiota extends beyond cancer type- and subtype-specific differences to spatial variations within the same tumor tissue. For example, state-of-the-art spatial profiling techniques have revealed significant microbial heterogeneity and region-specific distribution patterns in malignancies such as oral squamous cell carcinoma (OSCC) and CRC [98, 109]. Using 16S rRNA sequencing of dissected OSCC tissue samples, researchers [98] revealed that the outer tumor tissues were more enriched in *Fusobacterium*, *Neisseria*, *Porphyromonas*, and *Alloprevotella*, which contrasted with the inner regions where *Prevotella*, *Selenomonas*, and *Parvimonas* were more abundant. Although the authors did not perform mechanistic experiments, the following Kyoto Encyclopedia of Genes and Genomes analysis revealed that local TME factors, such as oxygen gradients, nutrient availability, and immune responses, might favor the localization of different microbial species [98]. Similarly, using advanced techniques including in situ spatial profiling and single-cell RNA sequencing (scRNA-seq), another elegant study demonstrated that tumor-resident bacteria in OSCC and CRC were prone to localize in less vascularized and highly immunosuppressive regions [109]. These study findings support the hypothesis that the spatial contribution of intratumoral microbes is determined by the local TME and emphasize the need to investigate how TME features shape microbial colonization and how these interactions affect cancer biology.

Despite microbial diversity across studies, certain taxa consistently appear in multiple cancers, suggesting potential shared roles in carcinogenesis. Oral commensals linked to periodontal infections, such as *Fusobacterium nucleatum*, *Porphyromonas* spp., and *Prevotella*, have been frequently detected in digestive cancers, including colorectal, esophageal, gastric, and pancreatic cancers, as well as breast and genital cancers [21, 26, 40, 63, 64, 67, 71, 72, 75, 76, 86, 92, 95–98, 105]. These taxa are often enriched in tumor tissues compared to matched healthy tissues, suggesting that they may share common colonization strategies within the TME and possibly contribute to cancer progression through similar mechanisms. Interestingly, prospective studies have shown that poor oral health is associated with increased cancer risk and a worse prognosis, suggesting a potential link between the oral microbiota and carcinogenesis [40, 110–112]. As such, these microbial signatures present opportunities for developing universal biomarkers for early diagnosis and prognostic assessment, as well as for designing microbial-targeted therapeutics that modify the tumor ecosystem to improve patient outcomes.

### Diagnostic and prognostic implications of intratumoral microorganisms

#### Tumor-specific microbial signatures for diagnosis

The heterogeneity of intratumoral microorganisms across cancer stages, genotypes, and phenotypes highlights their potential as novel diagnostic biomarkers [40, 113, 114]. Not surprisingly, numerous efforts have been focused on tumor types that are in close contact with the external environment, particularly those associated with mucosal surfaces that harbor significant microbial populations, such as CRC and OSCC. For example, in CRC, key taxa, including *Fusobacterium*, *Bacteroides*, *Parvimonas*, and *Prevotella,* shift significantly along the adenoma-carcinoma sequence and correlate with genetic alterations such as Kirsten rat sarcoma virus (*KRAS*) mutations and microsatellite instability (MSI) [72]. These associations underscore the potential of tumor-resident microbes as diagnostic biomarkers, not only for differentiating cancer stages but also for distinguishing cancer subtypes. Similarly, in OSCC, comparisons of precancerous, early-stage, and late-stage tumors revealed that *Capnocytophaga*, *Fusobacterium*, and *Treponema* were enriched in cancerous tissues, whereas *Streptococcus* and *Rothia* were more abundant in precancerous stages. Receiver operating characteristic (ROC) curve analysis revealed that *Capnocytophaga* and *Streptococcus* exhibited good diagnostic potential, with area under the ROC curve (AUC) values of 0.8103 and 0.7874, respectively [99].

The diagnostic potential of intratumoral microorganisms is not limited to tumors with mucosal origins. In breast cancer, multiple bacterial taxa, such as *Porphyromonas*, *Lacibacter*, *Ezakiella*, *Fusobacterium*, and *Stenotrophomonas,* were found to be significantly associated with cancer stage, subtype, receptor expression status, and metastatic potential after adjusting for confounders such as age and race, indicating that these bacterial taxa could be as independent impact factors [26]. Similarly, in hepatocellular carcinoma (HCC), distinct intratumoral microbial signatures were identified and found to correlate with clinical characteristics such as sex, cirrhosis grade, and tumor volume [83]. Although not yet clinically validated, the machine learning prediction model trained on either microbial class features (top 5 or all) or operational taxonomic unit (OTU) signatures (top 50 or all 3504 OTUs) derived from 16S rRNA sequencing achieved superior performance and accuracy in predicting HCC in both training and validation cohorts, with AUC values ranging from 0.939 to 1.000 [83]. These findings suggest the significant potential of the intratumoral microbiota as an independent diagnostic biomarker for cancer; however, further validation in larger and multicenter cohort studies is warranted.

#### Prognostic significance of tumor-resident microbiota for clinical outcomes

In addition to its diagnostic ability, the intratumoral microbiota also has prognostic value because of its potential impact on cancer progression and patient outcomes. In a multicenter retrospective cohort study of nasopharyngeal carcinoma, a higher intratumoral bacterial load was correlated with lower disease-free survival, distant metastasis-free survival, and overall survival rates [96]. Furthermore, a negative correlation was observed between the intratumoral bacterial load and immune cell infiltration-particularly CD8^+^ T cells and natural killer (NK) cells-along with the alteration of tumor proliferation features, underscoring the potential role of intratumor bacteria in modulating TIME and cancer cell phenotypes [96]. Given the high interindividual heterogeneity in amplicon sequence variants, the authors [96] suggested that the absolute bacterial load might serve as a simpler and more generalizable prognostic biomarker. However, tumor-resident microbes likely have diverse functions, promoting or inhibiting cancer progression, which could be masked if the bacterial load is used as the sole biomarker. Supporting this view, Sheng et al. [100] analyzed RNA-sequencing data from TCGA and demonstrated that immune-deficient and immune-enriched OV subtypes harbor distinct microbial communities, which were differentially associated with patient outcomes and acted as either risk or protective factors. Among these microorganisms, the authors identified 32 microbial species, including 26 risk-associated and 6 protective taxa, which together exhibited strong prognostic value for OV patients. Similarly, in pancreatic ductal adenocarcinoma (PDAC), patients with short-term survival (STS) and long-term survival (LTS) presented distinct intratumoral microbial profiles. Notably, the combination of LTS-enriched taxa (*Pseudoxanthomonas*, *Streptomyces*, *Saccharopolyspora*, and *Bacillus clausii*) accurately predicted LTS in both the discovery cohort (AUC = 97.51) and the validation cohort (AUC = 99.17) [103]. These findings suggest that incorporating microbial load and taxa-specific features into prognostic models could increase the accuracy of predictions and inform personalized treatment strategies.

Furthermore, studies underscore a close association between intratumoral microbes and TIME components, particularly T cells, macrophages, and NK cells, all of which play key roles in cancer immunity and patient outcomes [99, 102, 115–117]. Future research should be focused on elucidating the molecular mechanisms by which specific microbial taxa interact with the TIME and exploring microbial interventions to reprogram the TIME toward antitumor immunity. Such efforts could pave the way for incorporating microbiota-targeted strategies into cancer immunotherapy, ultimately improving outcomes across diverse cancer types.

#### Prognostic relevance of tumor-resident microbiota in metastasis

In addition to shaping the TIME, Fu et al. [29] demonstrated that tumor-resident intracellular bacteria-the predominant form of intratumoral bacteria-could promote metastasis in a mouse model of breast cancer, underscoring the ubiquitous role of these bacteria in cancer progression. Given that metastasis is a major risk factor for the survival of cancer patients, it is not surprising that metastasis-associated microorganisms hold significant prognostic potential. In support of this, in a study on cervical cancer, a machine learning model was utilized to accurately predict tumor metastasis on the basis of 15 differentially abundant microorganisms between the metastatic and nonmetastatic groups. Among these, the abundances of 5 taxa (*Robiginitomaculum*, *Klebsiella*, *Micromonospora*, *Microbispora*, and *Methylobacter*) were strongly associated with cervical cancer prognosis. The microbiome clusters defined by these 5 taxa corresponded with the differential expression of endogenous host genes, providing a robust predictive model for cancer prognosis [74]. These findings highlight the potential interplay of the intratumoral microbial composition with host genetic factors, collectively contributing to cancer progression and offering new avenues for prognostic biomarker development.

#### Intratumoral microbiota in therapy resistance and prognosis

The intratumoral microbiota has also been associated with cancer therapeutic resistance. In a study of cholangiocarcinoma, gemcitabine- and cisplatin-resistant tumors presented distinct microbial profiles, including enriched Gammaproteobacteria, which was correlated with tumor metabolic signatures, underscoring the functional role of the intratumoral microbiota in driving chemoresistance and its potential as a prognostic biomarker [69]. While retrospective studies provide valuable insights, the prognostic potential of the intratumoral microbiota is best validated through prospective research. For example, a 5 years prospective follow-up study on lung cancer identified 4 bacterial species *Marcescens, Actinomyces neesii, Enterobacter cloacae,* and *Haemophilus parainfluenzae*-detected after first-line treatment as significant prognostic biomarkers for 2 years survival. This model achieved an impressive accuracy rate of 90.7% [118], reinforcing the potential of intratumoral microbial features to predict treatment outcomes and long-term survival.

#### Context-dependent prognostic roles of specific microbes

Interestingly, the prognostic impact of certain microbes varies by cancer type, indicating that these tumor-resident microbes may function differently among tumor types and therefore cannot be generalized across cancers without considering the unique TME and host-microbe interactions specific to each cancer type. For example, *Fusobacterium nucleatum*, an important opportunistic pathogen, has been associated with a poor prognosis of CRC and esophageal cancer [71, 76] but appears to act as a favorable prognostic factor in anal squamous cell carcinoma and OSCC [66, 97]. Notably, the unexpected positive correlation between a higher load of *Fusobacterium nucleatum* and longer survival in OSCC patients contrasts with the established link between poor oral health and increased oral cancer risk. To explore this discrepancy, Neuzillet et al. [97] measured the expression of inflammatory and immune cell markers and reported an inverse association between *Fusobacterium nucleatum* loads and Toll-like receptor 4 (TLR4) expression as well as M2 macrophage abundance, which may partially explain this observation. However, the small sample size of the study necessitates validation of the findings. Moreover, a comprehensive investigation of the intricate interplay among *Fusobacterium nucleatum*, the TIME, and other microbial community members is warranted.

### Challenges and future directions in the application of microbiota-based biomarkers

To conclude, the evidence currently highlights that tumor tissues are heterogeneously enriched in microorganisms, and this enrichment varies across cancer stages and subtypes and is closely linked to prognosis. These findings underscore the potential of the intratumoral microbiota as a basis for developing novel diagnostic and prognostic biomarkers, as well as innovative therapeutic strategies targeting these microbial communities.

Despite recent advances, current studies investigating the intratumoral microbiota are subject to several methodological limitations. 16S rRNA sequencing, the most frequently applied technique in these studies, cannot identify microbes at the species level due to the highly conserved nature of the 16S rRNA gene across bacterial species, leading to ambiguous classifications. Additionally, in almost all of these studies, relative abundance analysis was used to compare microbial taxa between groups, assuming a constant total microbial load. This assumption may lead to biased interpretations, as tumor samples often have higher bacterial loads than paired control samples do [96]. In addition, variations in other taxa can influence the relative abundance of specific microbes, potentially resulting in false-positive findings. To address this, Barlow et al. [119] developed a quantification framework combining digital PCR with 16S rRNA gene sequencing to convert relative abundances into absolute measurements. Using this framework, they reported that although the relative abundance of *Akkermansia* in stool samples from mice fed a ketogenic diet was threefold greater than that in control mice, the absolute abundance remained constant, highlighting potential biases in relative abundance studies [119].

Moreover, some studies utilize reanalyzed data from cancer-focused databases, such as whole-exome sequencing and RNA sequencing, which are not optimized for microbial detection [64, 65, 74, 81, 93, 100]. Issues such as incomplete lysis of intratumoral microbes, interference from the host genome, and contamination from environmental microbes can compromise the reliability of these findings. To address these challenges and validate the relationship between the intratumoral microbiota and cancer, it is crucial to develop sequencing protocols specifically tailored for microbial detection. Research on microbial localization within tumors and functional validation through in vitro cell lines and mouse models are also essential.

Despite these limitations, techniques such as targeted PCR, microbial imaging, and culture-based methods have successfully confirmed the selective colonization of specific microbes in tumor regions [10]. These approaches enable precise examination of microbial localization, abundance, and functional roles within the TME. By integrating these advanced methodologies, future studies can deepen our understanding of the biological significance of intratumoral microbiota, shedding light on their roles in cancer progression and treatment responses.

## Origins and pathways of intratumoral microorganism colonization

**Fig. 1:**
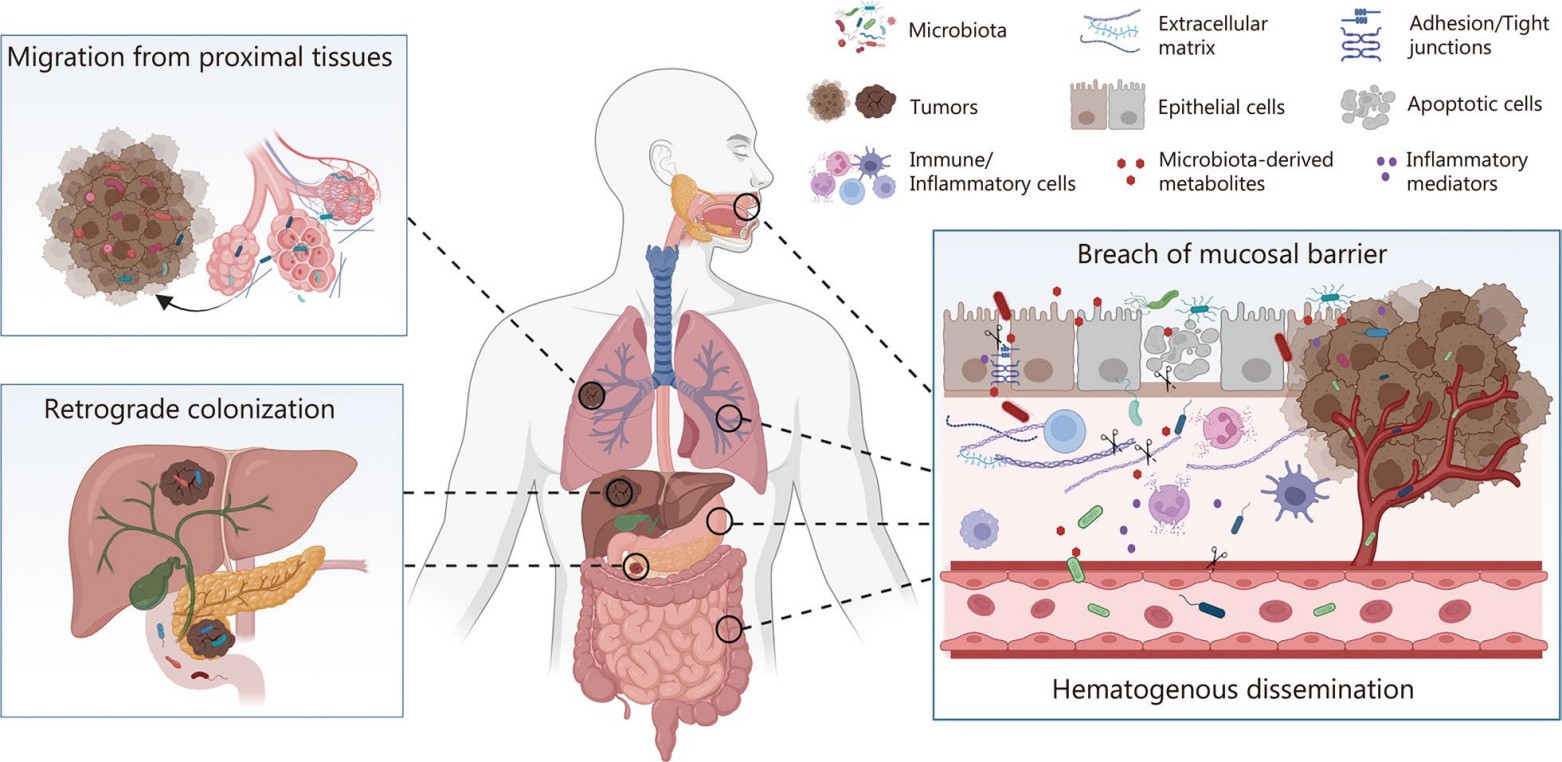
Proposed origins and dissemination pathways of intratumoral microbiota. Several potential routes through which microbiota colonize tumors have been proposed: 1) breach of mucosal barriers, where certain microbes disrupt epithelial integrity, allowing for microbial infiltration from mucosal surfaces into deeper tissues, promoting inflammation and cancer progression; 2) retrograde colonization, where the gut microbiota translocate to the pancreas and liver and colonize within pancreatic ductal adenocarcinoma and hepatocellular carcinoma tumors; 3) migration from proximal tissues, where microbes from nearby non-neoplastic tissues infiltrate tumors; and 4) hematogenous dissemination, where microbes occasionally penetrate the blood and colonize distant tumor sites via hematogenous transmission. These mechanisms highlight the complex interactions between microbiota and the tumor microenvironment, influencing tumor initiation, progression, and therapy resistance. Created with BioRender.com

Understanding how intratumoral microorganisms originate and colonize tumor tissues is crucial for elucidating their roles in cancer progression and therapy. Potential routes of bacterial infiltration include breaches in mucosal barriers and retrograde migration, migration from proximal tissues, and hematogenous dissemination, each offering unique insights into cancer biology (Fig. 1).

### Breaching of mucosal barriers and retrograde colonization

#### Breaching of mucosal barriers

Epithelial layers typically serve as barriers that prevent microbial invasion. However, when these barriers are compromised, bacteria and their byproducts can breach the “leaky epithelium” and penetrate deeper into tissues, fostering chronic inflammation and potentially contributing to tumor development [120]. A leaky epithelium could be particularly relevant for epithelial-derived carcinomas, which account for 90% of all human cancers [121]. Although such evidence is scarce, available data on host-microbe interactions may provide insights into this mechanism. For example, Grosheva et al. [122] conducted an imaging-based high-throughput screening of Caco-2 cells and identified several bacteria-derived substances, such as putrescine, acetyl-proline, and spermine, which disrupt the tight junctions (TJs) of intestinal epithelial cells. These disruptions are further aggravated by inflammatory cytokines such as tumor necrosis factor (TNF)-α and interleukin (IL)-1β , illustrating how microbial and host factors synergistically impair mucosal integrity [122]. In addition, pathogens such as *Shigella*, through the secretion of serine protease A, which disrupts epithelial actin dynamics, directly invade colonic epithelia and induce inflammation [123]. Interestingly, this process may be facilitated by the antimicrobial peptide human α-defensin 5, which binds to *Shigella* and promotes *Shigella* infection in a structure-dependent manner, implicating a complex interplay of bacteria and the host defense machinery [124].

Oral pathogens such as *Porphyromonas gingivalis*, *Tannerella forsythia*, and *Fusobacterium nucleatum* are also involved in the breach of mucosal barriers through various mechanisms. First, these microbes secrete proteases that degrade host proteins constitutively expressed in TJs, adhesive complexes, and the extracellular matrix, leading to disrupted epithelial integrity [125–128]. Second, they dysregulate host immune responses, triggering excessive inflammation that impairs mucosal repair [129, 130]. Third, their metabolites, such as SCFAs (e.g., butyrate), induce apoptosis of epithelial cells, further exposing the barrier structure [131, 132]. These mechanisms not only reveal the aggressive strategies of some pathogens but also facilitate secondary colonizers that further drive cancer progression. This process is conceptualized as the bacteria-driven passenger model [133], which may play a critical role in bacteria-induced carcinogenesis. For example, two well-recognized pathogens, toxigenic *Bacteroides fragilis*, and *H. pylori*, strongly associated with the development of colorectal and gastric cancers, may act as primary microbial drivers that induce inflammatory responses, genotoxic stress, and epithelial barrier disruption [51, 134]. Such alterations may subsequently create a permissive microenvironment that facilitates the colonization of opportunistic microbes and further promotes tumorigenesis.

Although these mechanisms are well documented in the context of microbial breaches of the epithelium, their relevance to the presence of tumor-resident microbiota remains unclear, partly because of the challenges associated with tracking microbial colonization during tumorigenesis. In most cases, the timing of bacterial colonization and its potential role as a causal factor in tumor onset remains debated. However, tumor model observations may offer valuable insights into these dynamics. For example, by using transgenic mice, Kostic et al. [135] demonstrated that *Fusobacterium* spp. promote intestinal tumorigenesis and proinflammatory response in *Apc*^*Min/*+^ mice, a model predisposed to colon cancer due to the *Apc* mutation, but not in *Il-10*^*−/−*^ and *T-bet*^*−/−*^*Rag2*^*−/−*^ mice, which represent inflammation-driven carcinogenesis. The phenomenon that *Fusobacterium* enhanced tumor development only in the presence of tumor-initiating mutations, but not in inflammation-prone models lacking such mutations, suggests that bacterial colonization may follow, rather than precede, somatic oncogenic events. This finding highlights the significant role of genetic mutations in creating a microenvironment conducive to microbial colonization. Despite these insights, the application of microorganisms to genetically predisposed models makes disentangling the effects of genetic factors from those of specific microbes in carcinogenesis difficult. To address this challenge, diverse tumor models-such as transgenic models, in situ cancer models, and chemical carcinogenesis models combined with 3D organoid systems that allow microbiota manipulation, could be instrumental. These approaches can help clarify the timing and causal relationships of microbial colonization in cancer progression and offer opportunities to explore novel intervention strategies.

#### Retrograde colonization

In addition to tumors originating from the mucosa, cancers such as PDAC and HCC, which seem isolated from external exposure, can interact with microbes from mucosal sites through retrograde colonization [85, 102, 136]. This mechanism is supported by clinical observations showing that the duodenal fluid of PDAC patients is enriched with PDAC-associated bacteria, such as *Bifidobacterium*, *Fusobacteria*, and *Rothia*, compared with that of samples from healthy pancreatic tissues or pancreatic cysts [137]. PDAC mouse models provide further valuable insights into these microbial dynamics during cancer progression. For example, Pushalkar et al. [102] demonstrated in PDAC transgenic mouse models that gut microbes can translocate to the pancreas and establish the PDAC microbiome. Using microbial labeling and repopulation experiments, they showed that bacteria such as *Enterococcus faecalis* and *Escherichia coli* (*E. coli*) could reach the pancreas via oral gavage and that microbes derived from the guts of invasive PDAC mice had a higher propensity to colonize the pancreas than those from WT donors [102]. Similarly, Aykut et al. [104] reported that fungal species introduced via oral gavage rapidly migrate to the pancreas and may evolve in response to PDAC progression, revealing a dynamic exchange among the gut, oral, and pancreatic microbiomes. Notably, sequencing data indicates significant structural differences between the PDAC intratumoral microbiota and paired gut microbiota [104]. These findings suggest that the PDAC microbiome is not a mere replica of the gut microbiome. Instead, gut bacteria colonizing PDAC tumors via retrograde infection may undergo further evolution influenced by the unique PDAC TME. The mechanisms underlying these microbiota evolutionary processes, as well as whether they involve interactions with pancreatic-resident bacteria, remain to be elucidated.

Retrograde colonization of tumors by microorganisms has also been observed in HCC, where *Mycoplasma hyorhinis* in the gastrointestinal tract can infect the liver through the oral-duodenal-hepatopancreatic ampulla route, promoting the initiation and progression of HCC [85]. This possibility is further supported by quantification studies showing significantly greater *Mycoplasma* loads in tumors, noncancerous adjacent tissues, and swabs from the bile duct, gallbladder, and duodenum than in peripheral blood and peritoneal fluid [85]. Notably, the latter two sample types presented microbial levels comparable to those of extraction controls, reinforcing the specificity of *Mycoplasma hyorhinis* colonization in tissues and ducts associated with HCC [85].

These studies highlight the critical role of retrograde infection in shaping the intratumoral microbiota, particularly in PDAC and HCC tumors. However, the potential involvement of retrograde colonization in other cancer types remains largely unexplored, presenting a promising area for future research. For example, tumors of the urinary and reproductive systems may harbor microbes originating from retrograde infections at their respective mucosal sites, as evidenced by the findings that the microbiomes of the urinary and reproductive tracts differ significantly among patients with and without urinary and reproductive cancers, as well as those with different cancer types and outcomes [138–141]. Although it remains unclear whether this distinct microbial signature is a cause or a consequence of cancer development, it is plausible that similar mechanisms of retrograde infection may contribute to their tumor-associated microbiomes. Investigating these routes of infection could provide valuable insights into the role of microbial communities in cancer development across diverse systems, thereby advancing our understanding of cancer pathogenesis and potential therapeutic strategies.

### Migration from proximal nonneoplastic tissues

Recent findings from studies employing advanced deep-sequencing methods have challenged the traditional belief that internal organs are sterile and revealed microbial communities in breast, bladder, and lung tissues with distinct structures compared with those in the oral cavity, gut, and skin [142–145]. These findings suggest that internal tissues harbor isolated microbial environments, raising questions about how microbes in nonneoplastic tissues might migrate to and influence cancerous tissues, thereby affecting cancer progression.

The evidence supporting this hypothesis includes the observed similarities in microbial composition between cancerous and adjacent noncancerous tissues, implying that the intratumoral microbiota may be derived from resident microbes within healthy tissues [10, 80]. However, alternative theories are also reasonable in that microbes in paracancerous regions could arise from the TME or represent ancestral species adapted to the unique cancer niche. Furthermore, studies on metastatic tumors have demonstrated that bacterial species found in primary tumors can persist through lymphatic and distant metastases [29, 71]. However, once microbes reach metastatic sites, the microbial composition of these sites appears to be shaped by the microenvironment of the distal organ. For example, Fu et al. [29] reported that the microbiota of primary breast tumors mirrored that of early lung micrometastases but differed significantly in later macrometastases. During the metastasis process, distinct bacterial clusters were identified: the presence of the “dominant cluster” was maintained from the primary site to early metastasis stages but declined in later stages; the “diminishing cluster” gradually decreased across all stages; and the “constituent cluster” was consistently observed at both primary and metastatic sites [29]. These findings suggest a dynamic and responsive interaction between the microbiota and the local environment of the tumor, reflecting an intricate balance between microbial adaptation and the host immune response.

Despite these insights, direct evidence for microbial migration from noncancerous tissues remains limited. This limitation highlights the need for a more precise characterization of microbial sources and dynamics. Future studies could have larger clinical sample sizes, and longitudinal observations could be incorporated to monitor microbial changes over time. Additionally, spontaneous mouse tumor models could be employed to trace microbial dynamics from early precancerous lesions through tumor progression. By comparing microbial profiles across precancerous tissues, tumor tissues, and adjacent noncancerous tissues, researchers could determine whether the intratumoral microbiota is derived from resident microbes or evolves in response to the TME. These investigations could provide valuable insights into the temporal and spatial patterns of microbial colonization and their roles in the initiation, progression, and metastasis of cancer.

### Hematogenous dissemination

In clinical studies, oral microbes such as *Fusobacterium* are frequently detected in various tumors, including colon and breast tumors, suggesting a potential hematogenous route for microbial transmission [67, 71, 146, 147]. In support of this hypothesis, bacteria introduced into the bloodstream were observed to successfully colonize TMEs [148, 149]. Additionally, tumor-associated bacteria such as *E. coli* have been shown to compromise vascular integrity in the gastrointestinal tract, facilitating their migration to the liver and the formation of premetastatic niches, which further underscores the potential of microbes to influence cancer progression through vascular interactions [150].

The phenomenon of bacteria being transported through the bloodstream remains a topic of debate because human blood is traditionally considered sterile, and the presence of pathogens in the blood, referred to as bacteremia, may lead to severe illnesses such as sepsis. However, studies have revealed that certain bacterial species, such as *Staphylococcus aureus*, *Chlamydia pneumoniae*, and *Streptococcus pneumoniae*, are unexpectedly prevalent in blood samples from healthy donors and are viable within erythrocytes, blood mononuclear cells, and neutrophils [151–154]. Findings from studies in which advanced sequencing techniques were employed further suggest the presence of multiple bacterial species in blood samples, raising the possibility of a blood microbiome [155–158]. However, these findings have been criticized due to the methodological limitations of the studies, such as inadequate decontamination, small sample sizes, and limited taxonomic resolution [159].

Recently, a study involving 9770 healthy individuals offered new insights. After implementing stringent decontamination protocols, researchers identified 117 microbial species in blood samples, and these microbes primarily originated from the gut, oral cavity, and genitourinary tract [160]. While the study revealed no consistent microbial co-occurrence patterns or evidence of core species-challenging the concept of a stable blood microbiome, the findings support a model in which microbes sporadically translocate from other body sites into the bloodstream. This sporadic translocation positions the bloodstream as a plausible pathway for the dissemination of intratumoral microbes.

Despite growing evidence for hematogenous dissemination, the underlying mechanisms remain poorly understood, partly owing to the typically low abundance of bacteria in blood and tissues, and the challenges in distinguishing the effects of these bacteria from those of other tumor-promoting factors. The development of advanced tools such as the StrainSifter pipeline represents a significant step forward. By enabling strain-level matching of bloodstream pathogens to their potential microbial reservoirs, this tool may provide critical insights into microbial translocation and tumor-associated infections [161]. Future studies leveraging such techniques could refine our understanding of microbial dissemination in cancer and facilitate the identification of microbial signatures predictive of metastasis or therapeutic resistance. Additionally, integrating these approaches with tumor models and longitudinal clinical data could uncover novel strategies for targeting hematogenous microbial dissemination in cancer.

## Selective colonization of tumors by microbes: mechanical insights into intracellular bacteria

The colonization of tumors by microbes occurs in two distinct stages, each reflecting a selective process. Initially, microbes colonize tumor tissues, exhibiting heterogeneity and region-specific patterns, as observed in OSCC, CRC, and PDAC [98, 109, 162]. This selective enrichment suggests that the TME, characterized by its unique pH, oxygen levels, nutrient availability, and immune landscape, creates conditions favorable for specific microbial communities [163–167]. The metabolic traits of tumors, including the Warburg effect, generate acidic and hypoxic conditions that may further promote microbial colonization [168, 169]. Additionally, genotypic variations in cancer cells may influence microbial preferences, reflecting a complex interplay between host genetics and microbial selection [14, 15, 21, 93]. In the second stage, microbes preferentially localize within cancer cells over healthy cells. This intracellular residency has been consistently identified across various tissues, including central nervous system tissues, skeletal structures, and mammary glands [10, 70, 170]. Intracellular bacteria, which constitute a predominant proportion of the intratumoral microbiota, leverage the intracellular environment for survival and proliferation, likely as a strategy to evade immune detection. These two stages, regional enrichments in the TME and intracellular localization within cancer cells, highlight the sophisticated interactions between microbes and tumors. Together, these findings underscore the need for further investigation into how these processes influence cancer progression and therapeutic outcomes.

Recent findings have revealed the profound impact of intracellular microbes on the behavior of cancer cells. For example, Fu et al. [29] demonstrated that breast cancer cells harboring intracellular bacteria exhibit increased resistance to biomechanical stresses, such as shear forces in circulation, due to cytoskeletal remodeling, potentially facilitating metastasis. Similarly, Galeano et al. [109] reported that intracellular bacteria significantly increase the migratory and invasive capacity of cancer cells, further supporting the role of intracellular bacteria in promoting metastasis. Notably, the potential of intracellular bacteria to promote metastasis appears to rely on their ability to penetrate and reside in cancer cells rather than specific bacterial species [29]. This phenomenon emphasizes the important contribution of bacterial invasive machinery and the intracellular lifecycle to cancer development and points to the opportunities of targeting bacterial invasion per se as a therapeutic strategy.

**Fig. 2:**
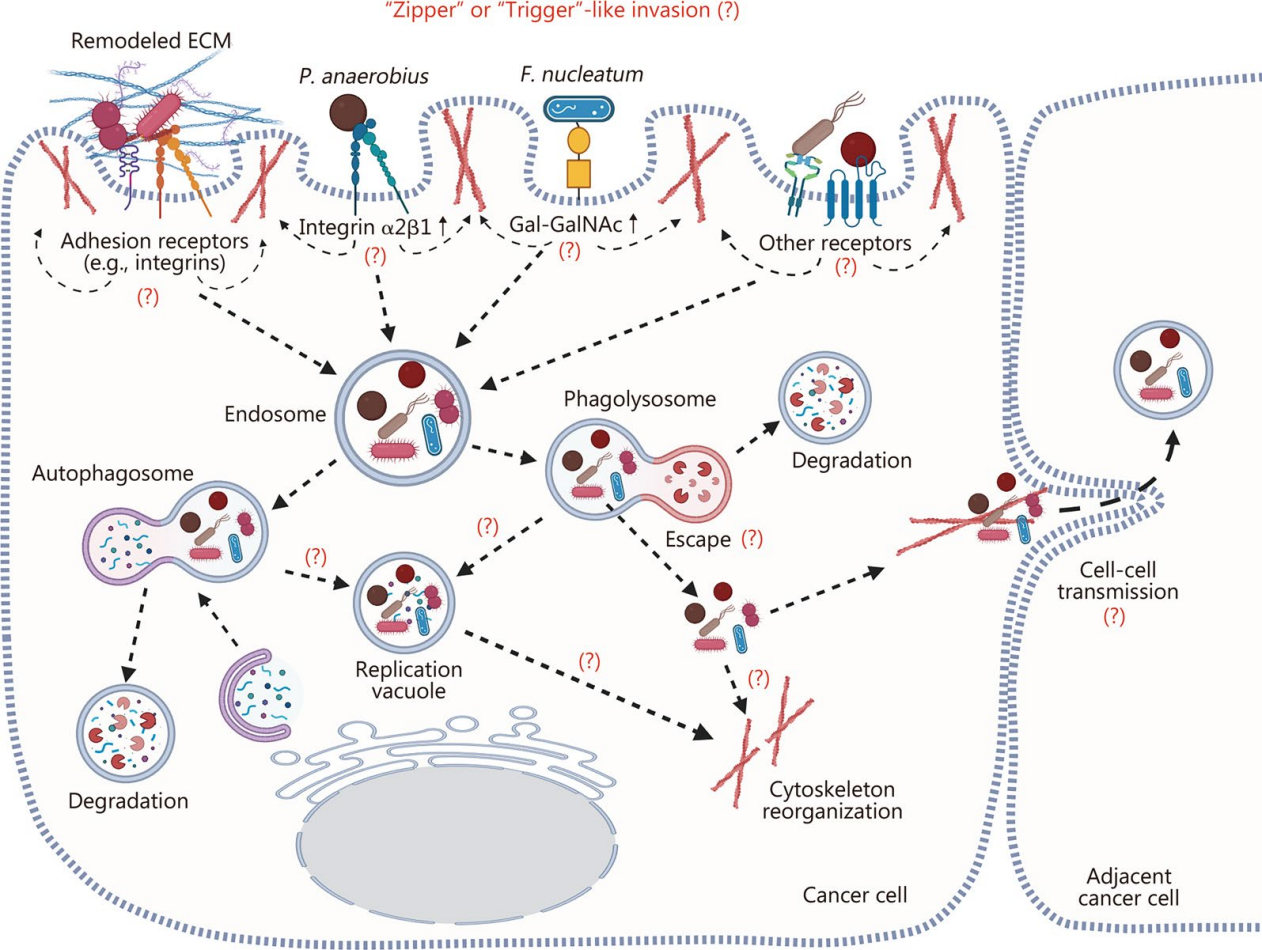
Proposed mechanisms of intracellular bacterial invasion and survival in cancer cells. The tumor-resident microorganisms may selectively recognize cancer cells by binding to remodeled extracellular matrix (ECM) components within the tumor microenvironment and to overexpressed cell surface receptors [e.g., integrin α2β1 for *Peptostreptococcus anaerobius,* and D-galactose-β (1–3)-N-acetyl-D-galactosamine (Gal-GalNAc) for *Fusobacterium nucleatum*]. This interaction facilitates the regulation of the host cytoskeleton, enabling “Zipper” or “Trigger”-like bacterial internalization. Once internalized, certain microbes interact with host organelles, manipulate processes such as phagosome maturation, autophagy, and cytoskeleton reorganization, and establish replication vacuoles or escape into the cytosol to evade lysosomal degradation. These mechanisms support bacterial survival, proliferation, and cell-to-cell transmission to adjacent cancer cells, ultimately driving cancer progression and resistance to therapy. The question marks “(?)” in this figure denote processes or mechanisms that have been implicated in the context of pathogen infection in normal cells but remain unexplored or not fully elucidated in the context of cancer. These areas, including potential bacterial invasion strategies, intracellular survival pathways, and mechanisms facilitating intercellular transmission, warrant further investigation. Created with BioRender.com

In subsequent sections, we critically review the current knowledge on microbial incursion into cancer cells and their interaction with host cells, as summarized in Fig. 2. This exploration is vital for advancing cancer pathology and developing therapeutic modalities aimed at combating these microbial-tumor interactions.

### Mechanical insights into tumor microbial invasion

The mechanisms by which microbes invade cancer cells remain largely speculative, but insights can be drawn from studies of pathogenic bacteria, such as *Listeria monocytogenes* and *Salmonella typhimurium*, interacting with normal host cells [171–176]. These pathogens utilize two main strategies for invasion: the “zipper” mechanism, where direct interactions with host cell receptors facilitate uptake, and the “trigger” mechanism, involving cytoskeletal rearrangements induced by a type III secretion system [177, 178]. These models provide a foundation for understanding how intratumoral bacteria may adapt to the TME, although this remains an underexplored area.

The tumor ECM, which is altered by cancer-related changes in laminin, fibronectin, and glycoproteins, may serve as a novel substrate for microbial adhesion, leveraging the frequently observed capability of bacteria to bind to ECM components to facilitate their invasion [179, 180]. For example, *Staphylococcus aureus* binds to fibronectin through its adhesion proteins fibronectin-binding proteins A and B, forming a tripartite complex with integrins that facilitate invasion [181]. Similarly, *Campylobacter jejuni* adheres to fibronectin via *Campylobacter* adhesion to fibronectin (CadF) and fibronectin-like protein A (FlpA), a process essential for its internalization [182]. Although direct evidence linking these mechanisms to cancer is limited, it is plausible that tumor ECM remodeling, driven by matrix metalloproteinases (MMPs), creates opportunities for microbial colonization. Furthermore, MMP activity not only modifies ECM components but also releases matricryptins, which could serve as additional binding sites for tumor-associated bacteria [183–185].

In addition to ECM changes, the overexpression of specific receptors on cancer cells provides another avenue for selective bacterial colonization. Studies have confirmed that the overexpression of receptors on cancer cells, such as D-galactose-β(1–3)-N-acetyl-D-galactosamine (Gal-GalNAc) and α2β1 integrin, facilitates the colonization of *Fusobacterium nucleatum* in CRC and *Peptostreptococcus anaerobius* in breast cancer, respectively [67, 186, 187]. These data offer insights into selective bacterial invasion into cancer cells over healthy cells and indicate that the overexpression of host adhesion molecules may be an essential mechanism for structured intratumoral colonization by microbes. Future research into tumor-specific receptors is critical for understanding how microbial attachment influences cancer biology.

Once bacteria attach, internalization is accompanied by cytoskeletal rearrangements leading to phagosome or endosome formation. Pathogens exploit host actin-dependent mechanisms to facilitate this process. For example, *Campylobacter jejuni* activates the small GTPase Rac1 signaling pathway and recruits the autophagy-related protein LC3 to aid in bacterial engulfment [188]. Similarly, focal adhesion kinase (FAK) interacts with cortactin to regulate actin dynamics, promoting the internalization of pathogens such as *Staphylococcus aureus* via integrin α5β1 engagement [189]. Notably, the inhibition of FAK significantly reduces bacterial internalization rates [190, 191], underscoring the importance of FAK in bacterial invasion. While direct evidence linking FAK activity to intratumoral bacterial invasion is scarce, tumor-associated bacteria such as *Porphyromonas gingivalis* have been shown to activate FAK during invasion into healthy epithelial cells, and further investigation into similar mechanisms in cancer contexts is warranted [192].

Although these insights into bacterial invasion are derived from pathogen models, researchers do demonstrate that cancer cell actin machinery dynamics are altered and are associated with cancer progression [193, 194]. This hypothesis is therefore intriguing for understanding the interactions between intratumoral microbes and cancer cells. Key molecules in these pathways, such as integrins, growth factor receptors, small GTPases, and FAK, could serve as modulators of tumor bacterial invasion or as therapeutic targets. Investigating these mechanisms will expand our understanding of cancer biology and provide new avenues for cancer therapy.

### Intracellular niche of tumor-resident bacteria: implications of host machinery

In cancer research, the intracellular behavior of tumor-resident bacteria remains poorly understood and represents a major gap in our knowledge of tumor-microbe interactions. Findings from previous studies have indicated that intracellular pathogens, including bacteria, viruses, and parasites, evolve sophisticated mechanisms to manipulate the organelles of host cells and create an environment that is favorable for their survival and replication [195–199]. For example, *Salmonella* utilizes its secretion systems to inject effector proteins into host cells, altering phagosome maturation to create *Salmonella*-containing vacuoles suitable for survival [196]. In another example, *Legionella* manipulates the endoplasmic reticulum (ER) and Golgi membrane trafficking to establish replication vacuoles [197]. In addition, these intracellular pathogens may evade lysosomal degradation using the cell’s defense mechanisms or by repurposing them, often through intricate and precise strategies [198, 199]. Some bacteria, such as *Coxiella burnetii*, persist in lysosome-derived vacuoles [200–202], whereas others, such as *Orientia tsutsugamushi*, flourish freely in the cytosol with a dual lifestyle distinct from their extracellular counterparts [203]. Their exit mechanisms are also intricate, from secretion via vesicles to cytoskeletal-mediated transfer to neighboring cells [204], offering valuable insights into intratumoral microbiota research.

The interaction of intracellular pathogens with host organelles is important since the manipulation of host organelles can lead to significant alterations in host cell physiology. For example, the ER is often targeted by viruses to facilitate their replication, as observed with the hepatitis C virus [195]. This manipulation may disrupt normal cellular functions by interfering with host metabolism, protein folding, and lipid synthesis, thereby inducing pathology. In another example, Qiao et al. [85] reported that intracellular *Mycoplasma hyorhinis* induces mitochondrial fission through the suppression of mitochondrial fusion protein (*MFN*)*1* mRNA in an m^6^A-dependent manner, leading to pathological polyploidization and promoting HCC initiation and progression. This study highlights the importance of intratumoral microbes, and their predominant intracellular form in cancer progression through interaction with the host cellular compartment, and further studies on other intratumoral microbial species are warranted. Given this consideration, techniques such as advanced high-resolution fluorescence microscopy would allow researchers to visualize the colocalization of intracellular microorganisms with host organelles in real time, providing insights into how these interactions affect cancer progression [205, 206].

Autophagy is another important biological process that maintains cellular homeostatic equilibrium by recognizing and degrading damaged organelles and misfolded proteins, as well as recycling intracellular components, to provide nutrients and energy under conditions of starvation or cellular stress [207]. In the context of pathogen infection, autophagy is intricately modulated, where it plays a dual role in either eliminating intracellular microbes or inadvertently supporting their survival. Some microbes, such as *Pseudomonas aeruginosa* and *Salmonella enterica*, are eliminated via autophagosomes that mature into autolysosomes [208, 209]. However, other bacteria, such as *Listeria monocytogenes* and *Porphyromonas gingivalis*, evade or hijack autophagy machinery to establish nutrient-rich niches for replication in macrophages and endothelial cells, respectively [210, 211]. Moreover, pathogens such as uropathogenic *E. coli* (UPEC) further exploit autophagy, specifically ferritinophagy, a subtype of autophagy that degrades ferritin to release iron, which the intracellular UPEC uses as a nutrient for its survival and persistence in autophagosomes and lysosomes, as demonstrated in bladder epithelial cells [212]. These study findings emphasize the complex interplay of intracellular microbes with host autophagy pathways, which may play significant roles in modulating both the lifecycle of intracellular microbes and host cell physiology. In cancer, this interplay may play a key role in cancer progression and therapeutic response, considering the dual role of autophagy in either suppressing carcinogenesis or promoting cancer cell survival under stress conditions (e.g., hypoxia, nutrient deprivation, and chemotherapy) [147, 213, 214]. The impact of this interplay varies across cancer stages and subtypes, and further study is warranted.

The cytoskeleton plays a critical role in intracellular bacterial trafficking and cell–cell transmission, as shown by *Porphyromonas gingivalis* infection of gingival epithelial cells [215]. Findings from an increasing number of studies indicate that intracellular bacteria within cancer cells also actively influence host cellular actin reorganization, with some manipulating the Ras homolog family member A (RhoA)-Rho-associated protein kinase (ROCK) signaling pathway to prevent apoptosis and increase survival, a phenomenon observed by Fu et al. [29]. Given these findings, it is crucial to explore whether these strategies are universal across bacterial species within healthy and malignant cells, and the significance of these strategies in cancer progression, such as metastasis, which remains an open question in the field.

To conclude, the selective colonization of tumors by microbes, particularly their intracellular localization, represents a sophisticated interaction with cancer cells. Unlike classic pathogen infections, which often trigger cell death, intratumoral bacteria appear to coevolve with tumors, supporting cancer cell survival, migration, and therapy resistance. Understanding the mechanisms underlying microbial invasion, intracellular persistence, and interactions with host cellular machinery will provide critical insights into cancer biology. This knowledge holds significant potential for developing innovative cancer therapies targeting these microbial interactions within the TME.

## Mechanistic insights into intratumoral microbes and their impact on tumor dynamics

**Fig. 3:**
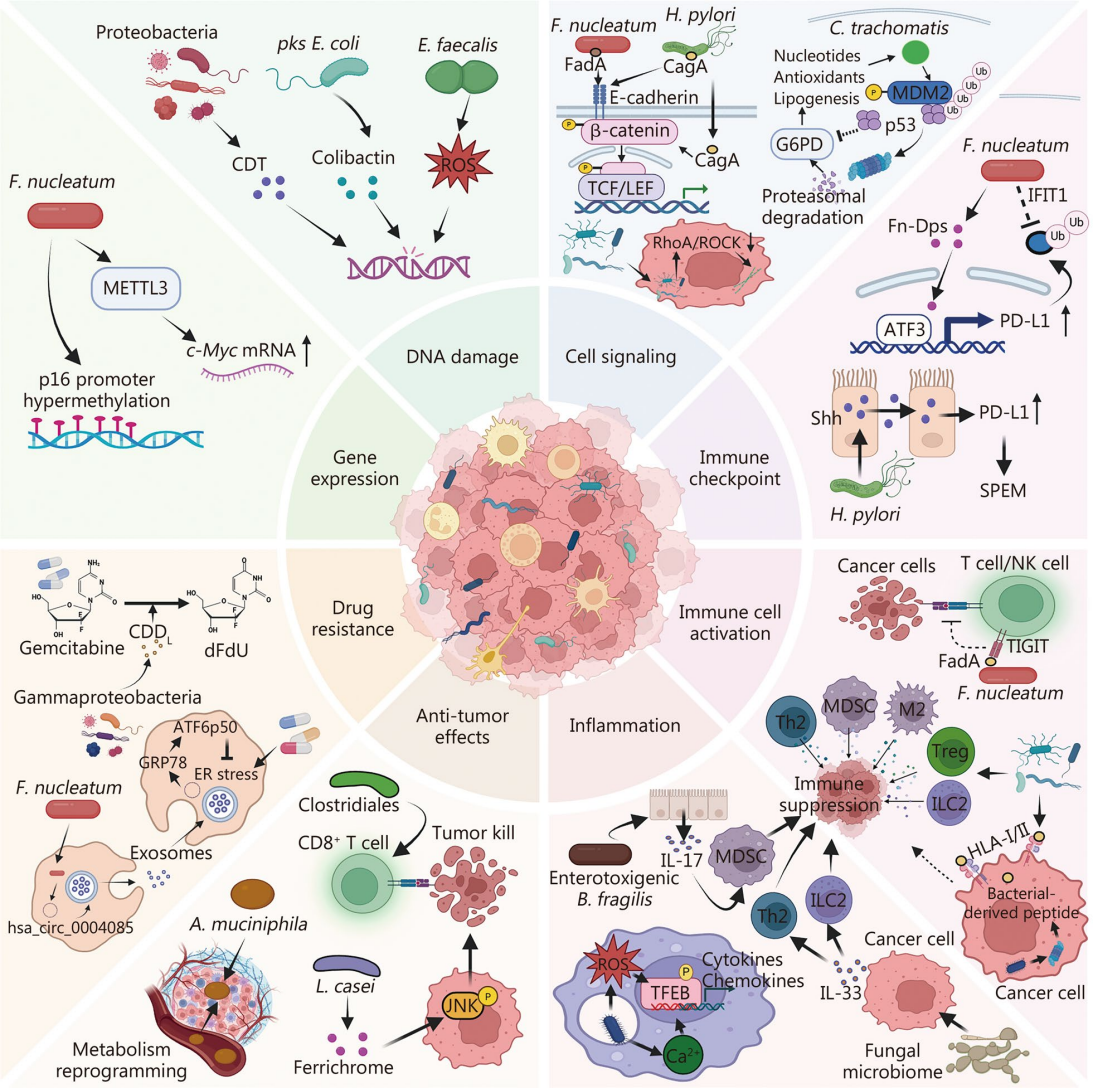
Proposed roles of intratumoral microbiota in cancer progression: mechanisms of DNA damage, immune modulation, and drug resistance. ATF6 activating transcription factor 6, CagA cytotoxic associated gene A, CDD_L_ the long isoform of cytidine deaminase, CDT cytolethal distending toxin, dFdU 2’-Difluorodeoxyuridine, ER endoplasmic reticulum, Fn *Fusobacterium nucleatum*, G6PD glucose-6-phosphate dehydrogenase, GRP78 glucose-regulated protein 78, HLA human leukocyte antigens, IFIT1 interferon-induced protein with tetratricopeptide repeats 1, IL interleukin, ILC2 group 2 innate lymphoid cell, JNK c-Jun N-terminal kinase, MDSC myeloid-derived suppressor cell, METTL3 methyltransferase-like protein 3, PD-L1 programmed death-ligand 1, RhoA Ras homolog family member A, ROCK rho-associated protein kinase, ROS reactive oxygen species, Shh sonic hedgehog, SPEM spasmolytic polypeptide-expressing metaplasia, TFEB transcription factor EB, TIGIT T cell immunoglobulin and immunoreceptor tyrosine-based inhibitory motif domain, Treg regulatory T cell, FadA *Fusobacterium* adhesin A, TCF T cell factor, LEF lymphoid enhancer-binding factor, MDM murine double minute, NK natural killer, Th T helper, c-Myc cellular myelocytomatosis oncogene, mRNA messenger RNA, ATF activating transcription factor, GRP glucose-regulated protein, Dps DNA-binding protein from starved cells, ATF6p50 activating transcription factor 6, 50 kDa cleaved form. Created with BioRender.com

Although numerous studies have linked intratumoral microbiota to cancer characteristics, the causal relationship remains under debate. Nonetheless, emerging evidence suggests that tumor-resident microbes may contribute to cancer progression and prognosis through diverse mechanisms, reviewed in recent literature [216–218]. Here, we provide an overview of their genomic and immune-modulatory impacts on tumor dynamics and therapeutic responses as summarized in Fig. 3.

### Impact of host cell genetic and epigenetic alterations by intratumoral microbes

Evidence suggests that intratumoral microbes induce substantial genomic and epigenetic alterations in host cells, contributing to carcinogenesis and cancer progression. Notably, certain strains of *E. coli* produce colibactin, which promotes genomic instability in gastric cancer in the presence of *pks* pathogenicity islands [219]. Similarly, Proteobacteria secrete cytolethal distending toxins that impair DNA repair mechanisms, further compromising genomic integrity [220]. Additionally, *Enterococcus faecalis*, a microbe known for its production of reactive oxygen species, induces DNA damage, providing another mechanism that intratumoral microbes may use to affect cancerous genetic changes [221]. These findings highlight the diverse mechanisms through which intratumoral microbes influence tumorigenesis and accelerate cancer progression.

Epigenetically, *Fusobacterium nucleatum* has been implicated in the hypermethylation of tumor suppressor gene promoters, such as *CDKN2A* (p16), in CRC, leading to gene silencing and cancer progression [222]. In esophageal squamous cell carcinoma, *Fusobacterium nucleatum* increases metastatic potential by upregulating the expression of methyltransferase-like 3, a key m^6^A methyltransferase. This, in turn, increases the expression of oncogenes, including *c-Myc*, as demonstrated in both in vitro and in vivo studies [223]. These findings exemplify how intratumoral microbes modulate host epigenetics, warranting further exploration of other taxa and their roles in cancer biology.

### Modulation of host oncogenic pathways by intratumoral microbes

Moreover, multiple oncogenic pathways may be modulated by intratumoral microbes. For example, the Wnt/β-catenin pathway, which is frequently dysregulated in various cancers, is modulated by bacterial proteins such as CagA from *H. pylori* and *Fusobacterium* adhesin A (FadA) from *Fusobacterium*, both of which act as potent modifiers of this pathway [224–226]. In addition, downregulation of the tumor suppressor p53 has been reported in infections with *Shigella*, *Helicobacter*, and *Chlamydia* [227–229]. Notably, *Chlamydia*, a classic obligate intracellular bacterium, suppresses p53 expression to ensure its persistence by redirecting host metabolism through the regulation of the pentose phosphate pathway [228]. This suppression not only promotes bacterial survival but also strengthens the Warburg effect in cancer cells, further supporting tumor metabolism and survival [228]. While validation of this mechanism in the context of cancer is required, the mechanism suggests a coevolutionary relationship between cancer cells and intratumoral microbes in which microbes and tumors mutually benefit in terms of survival and progression, respectively.

Intriguingly, the influence of tumor-resident microbes extends beyond infected cancer cells to neighboring cells through extracellular mechanisms. For example, in CRC, *Fusobacterium nucleatum* can invade cancer cells and promote pre-ephrin type-B receptor 2 (EPHB2) reverse splicing into hsa_circ_0004085, a circular RNA for which its expression is negatively correlated with patients’ response to the chemotherapeutic drugs oxaliplatin and 5-fluorouracil (5-FU), through the regulation of heterogeneous nuclear ribonucleoprotein (hnRNP) L [230]. Concurrently, hnRNP A1 is also induced, which binds to and facilitates the packaging of hsa_circ_0004085 into exosomes [230]. These exosomes are subsequently delivered to recipient cells and relieve ER stress through the regulation of glucose-regulated protein 78 and activating transcription factor 6α, resulting in chemoresistance [230]. These findings provide critical insights into how the intratumoral microbiota, despite its low biomass, can exert profound effects on cancer progression through intercellular communication.

### Impact of intratumoral microbes on tumor immunity

The profound immunomodulatory roles of microbial communities within tumors are increasingly recognized as pivotal factors influencing both cancer progression and the efficacy of immunotherapies. These microorganisms significantly affect innate and adaptive immune cells across various cancers, including colorectal, lung, pancreatic, liver, gastric, breast, ovarian, and oral cancers [99, 100, 102, 115, 116, 149, 166, 231]. By secreting inflammatory mediators and reprogramming the TIME, these microorganisms orchestrate specific immune responses that drive cancer onset and progression.

In a murine model of CRC, enterotoxigenic *Bacteroides fragilis* was shown to induce submucosal IL-17 secretion, promoting the recruitment and activation of myeloid-derived suppressor cells (MDSCs) within the TME [232]. This interaction not only suppresses immune effector cells but also activates proliferative and proangiogenic pathways, thereby driving tumor growth [232]. Similarly, in a murine lung cancer model, commensal bacteria stimulate the Myd88-dependent production of IL-1β and IL-23 from myeloid cells, which then trigger the proliferation and activation of Vγ6^+^Vδ1^+^ γδ T cells. These T cells produce IL-17 and other effector molecules, promoting both inflammation and cancer cell proliferation [233]. Moreover, in PDAC, the oncogenic mutation *Kras*^*G12D*^ promotes IL-33 expression, which is driven by the intratumoral fungal mycobiome. This leads to the recruitment and activation of protumorigenic Th2 cell and group 2 innate lymphoid cell (ILC2), which foster an immunosuppressive and inflammatory TME. Therefore, targeting IL-33 or associated fungal components may be a promising strategy to curb inflammation and improve therapeutic outcomes [234].

Complementing these findings, innate immune cells, including MDSCs, macrophages, neutrophils, and NK cells, are increasingly recognized as the key targets of microbial modulation. Emerging evidence highlights the significant role of tumor-resident microbes in modulating the activity and function of these cells, thereby influencing tumor immunity and progression. For example, in PDAC, the presence of tumor-resident bacterial species is correlated with an increase in MDSCs and a reduction in M1 macrophage differentiation [102]. This shift contributes to an immunosuppressive TIME characterized by diminished T-cell activity, ultimately impeding effective antitumor immune responses. In CRC, the abundance of *Fusobacterium nucleatum* is associated with increased infiltration of immunosuppressive M2-polarized macrophages. This alteration fosters a tumor-permissive TIME, thereby facilitating cancer progression [116].

Beyond innate immunity, tumor-resident microbes exert profound effects on adaptive immune responses, particularly those mediated by T cells, leading to tumor immune evasion. In gastric cancer, specific microbial populations reduce the number of CD8^+^ memory T cells within the TME, contributing to immune evasion [115]. In addition to the dynamic interplay within the TME, shifts in the intratumoral microbiota across disease stages in OSCC correlate with significant changes in T-cell infiltration and functionality, potentially affecting clinical outcomes [99]. In breast cancer, Chen et al. [231] utilized integrated bulk and scRNA-seq to reveal significant associations between intratumoral microbial signatures and host metabolic pathways. Notably, several metabolism-related microbes showed strong correlations with regulatory T cells and activated NK cells, suggesting their potential role in modulating TIME through metabolic-immune interactions.

Emerging mechanistic insights have further revealed how specific microbial species contribute to immune suppression. Some bacterial species directly interact with immune cells, thereby regulating immune function. For example, the *Fusobacterium* adhesin protein 2 (Fap2) of *Fusobacterium nucleatum* binds to T cell immunoglobulin and immunoreceptor tyrosine-based inhibitory motif domain, a receptor on T cells and NK cells, inhibiting their cytotoxicity and proliferation, thereby fostering an immunosuppressive TME [117]. Moreover, intratumoral microbes influence tumor immunity through the modulation of immune checkpoints. *Fusobacterium nucleatum*, for example, suppresses T-cell activity by inducing the expression of programmed death-ligand 1 (PD-L1) on cancer cells [235, 236]. This suppression may be achieved through the secretion of the virulence factor *Fusobacterium nucleatum*-derived DNA-binding protein from starved cells (Fn-Dps), which binds to activating transcription factor 3, thereby promoting *PD-L1* transcription [237]. Interestingly, similar regulatory effects on immune checkpoint expression have been consistently reported for other microbial species, such as *H. pylori*, *Porphyromonas gingivalis*, and *Prevotella intermedia* [55, 238, 239], which highlights a common mechanism through which tumor-resident microbes promote a suppressive TIME. This intricate interplay could significantly affect the efficacy of anticancer immunotherapy, particularly therapies that target immune checkpoints [19, 27, 240–242]. Further understanding of these interactions could be pivotal in increasing the efficacy of immunotherapeutic strategies in cancer treatment.

Intriguingly, the interaction between *Fusobacterium nucleatum* and tumor-infiltrating lymphocytes appears predominantly in tumors with high-MSI but not in those with low-MSI [243]. This observation underscores the complex interplay among bacteria, tumor immune dynamics, and genetic factors within the tumor, which may impact the efficacy of therapeutic strategies targeting these pathways.

In addition to genetic and immune factors, the spatial heterogeneity of intratumoral microbial colonization also contributes to shaping tumor immune responses. A recent spatially resolved single-cell study by Galeano et al. [109] demonstrated that intratumoral bacteria preferentially colonize immunosuppressive microniches within CRC and OSCC tumors, characterized by CD66b⁺ neutrophil accumulation, T-cell exclusion, and increased expression of immunosuppressive molecules, including programmed cell death protein 1 (PD-1), cytotoxic T-lymphocyte-associated protein 4, and arginase 1. Through INVADEseq-based scRNA-seq, the authors further revealed that bacteria-positive epithelial cells exhibit increased expression of proinflammatory chemokines, such as C-X-C motif chemokine ligand (*CXCL*) *10*, *CXCL11,* C–C motif chemokine ligand (*CCL*) *4*, and *CCL3*, alongside matrix-remodeling enzymes, including *MMP9* and *MMP3* [109]. These transcriptional changes were accompanied by a shift from a proliferative state toward an inflammatory and migratory state. In immune cell compartments, particularly macrophages, bacterial colonization induces the upregulation of the expression of interferon-stimulated genes (e.g., *GBP1* and *IFITM1*), cytokine-encoding genes such as *IL1B*, *IL6*, and *IL10*, and chemokine-encoding genes such as *CCL2*, *CCL4*, *CCL8*, *CCL7*, *CXCL1*, and *CXCL10*, which is consistent with polarization towards an immunosuppressive phenotype [109]. These results highlight a dual effect whereby intratumoral bacteria modulate both epithelial and innate immune cells to create a tumor-permissive and immune-excluded microenvironment. Such modulation may support bacterial persistence while concurrently suppressing antitumor immunity [244].

Finally, the discovery that tumor-resident microbes predominantly colonize the cytoplasm of cancer cells or reside within tumor-infiltrating immune cells raises critical questions about how these intracellular microorganisms influence the TIME. A notable study by Kalaora et al. [245] utilized 16S rRNA sequencing and human leukocyte antigen peptidomics to examine melanoma samples and revealed that peptides derived from intracellular bacteria can be presented by cancer cells, eliciting an immune response. In addition to antigen presentation, another critical pathway involves macrophage activation during bacterial phagocytosis. Najibi et al. [246] identified the NADPH oxidase (NOX/PHOX)-CD38-nicotinic acid adenine dinucleotide phosphate-transcription factor EB (TFEB) axis as a key regulator in this process. The activation of TFEB and transcription factor E3 leads to the induction of proinflammatory cytokines such as IL-6 and TNF-α, potentially driving protumoral immunity. These studies provide a nuanced view of the complex interactions of intracellular microbes in cancer immunology and highlight potential mechanisms through which intracellular microbes influence tumor progression. Such insights are crucial for the development of targeted therapies that can more effectively manipulate the TME for therapeutic benefit. Further research in various cancer types is warranted to explore the broader implications of these findings and to refine strategies for leveraging these mechanisms in cancer therapy.

### Potential antitumor effects

In addition to the recognized oncogenic roles, certain commensal microbiota exhibits antitumor effects, highlighting its dual impact on cancer progression. For example, early-life exposure to microbes reduces colonic tumorigenesis by modulating immune cells within the tumor environment [247]. Similarly, specific strains of *Clostridiales*, often depleted in CRC patients, demonstrate potent antitumor immune responses by interacting with host immune pathways [248]. In addition, gut bacteria such as *Akkermansia muciniphila* reprogram tumor metabolism and interact with intratumoral microbes to suppress tumor growth [249]. Furthermore, in CRC, *Lactobacillus casei* ATCC334 produces ferrichrome to kill cancer cells through the activation of c-Jun N-terminal kinas [250]. However, these findings were based on in vitro experiments, and whether similar mechanisms occur in vivo remains uncertain.

While many *Lactobacillus* species are considered probiotics, not all exhibit antitumor effects. Multiple cancer types are enriched in *Lactobacillus* species, including esophageal, gastric, lung, and cervical cancers, with some identified as predictive biomarkers of poor outcomes [64, 77, 79, 95, 251]. In cervical cancer, *Lactobacillus iners* rewires tumor metabolism by providing L-lactate, which induces chemoradiation resistance and promotes tumor growth in organoid models [251]. Notably, this effect is specific to *Lactobacillus iners*, as *Lactobacillus crispatus*, which produces D-lactate rather than L-lactate, does not exhibit similar effects [251]. Moreover, the *Lactobacillus iners* strains within cervical tumors were distinct from noncancer strains and carried additional genes for lactose utilization, which may contribute to increased production of L-lactate [251]. These study findings add complexity to the association of intratumoral microbes with tumor dynamics, suggesting strain-specific effects for which further investigation is merited.

The intricate interactions between intratumoral microbiota and cancer cells underscore the multifaceted roles of microbiota in cancers that merit further exploration. By modifying oncogenic pathways and impacting immune surveillance, intratumoral oncomicrobes facilitate cancer progression and affect therapeutic interventions’ outcomes. While current research has started to uncover the broad effects of intratumoral microbes, the precise molecular dialogues between these microbes and cancer cells remain poorly defined. This lack of detailed knowledge constrains the potential to develop microbial-targeted therapies to enhance cancer treatment efficacy.

## Emerging strategies to target the intracellular tumor-associated microbiota and exploit microbial systems for cancer therapy

### Antibiotics: opportunities and challenges

The recognition of tumor-resident microbes as key players in cancer progression and therapeutic efficacy is driving the development of innovative strategies aimed at eradicating these microorganisms to combat cancer. Among these strategies, antibiotics have been the primary focus. Oral administration of the antibiotic metronidazole significantly reduced the *Fusobacterium* load in a mouse xenograft model, inhibiting cancer cell proliferation and tumor growth, which highlights the potential of antimicrobial interventions as viable anticancer therapeutic strategies [71].

Prophylactic antibiotics are frequently administered to cancer patients during surgery, effectively preventing infection at the surgical site and reducing infectious complications in cancer types such as breast, colorectal, and liver cancers [252–254]. However, the use of antibiotics in cancer therapy is not without concern. One major issue is nonselective bacterial killing by antibiotics, which may lead to systemic microbiota dysbiosis and adverse side effects [255, 256]. Given the diverse roles of the human microbiota in cancer, these side effects may have detrimental consequences [257–260].

In hematological malignancies, particularly B-cell lymphoma, the adverse effects of antibiotics on cancer outcomes are increasingly being recognized. Pretreatment with broad-spectrum antibiotics, recognized as “high-risk” antibiotics, 3 weeks before CD19-targeted chimeric antigen receptor (CAR)-T-cell therapy is associated with accelerated disease progression and reduced patient survival. This finding was consistently observed in two independent cohorts and was associated with a disruption of the gut microbiota structure induced by antibiotic treatment, resulting in the loss of beneficial taxa such as *Bifidobacterium longum* and *Akkermansia muciniphila* and impaired immune modulation [257]. Consistent with this finding, another cohort study of B-cell lymphoma and leukemia patients revealed that exposure to antibiotics, particularly piperacillin/tazobactam, meropenem, and imipenem/cilastatin, 4 weeks before CAR-T-cell therapy was associated with worse survival and greater neurotoxicity. This phenomenon was associated with structural changes in the gut microbial community and significantly altered metabolic pathways, which may play key roles in the modulation of the host immune response [260].

These adverse effects also extend to non-hematological malignancies. In a murine orthotopic breast cancer model, the administration of a cocktail of antibiotics, including vancomycin, neomycin, metronidazole, amphotericin, and ampicillin significantly accelerated breast cancer progression [259]. This acceleration could be attributed to the loss of gut commensal bacterial taxa that are associated with the accumulation of mast cells in the tumor stroma [259]. Notably, reintroducing one missing taxon, *Faecalibaculum rodentium*, via oral gavage restored tumor growth to control levels, supporting the idea that antibiotics can modulate the gut microbiome to influence tumor dynamics [259]. In clinical cohorts involving patients with non-small cell lung cancer, melanoma, and other tumor types recruited from routine clinical practice, Pinato et al. [261] revealed that pretreatment with antibiotics before immunotherapy was associated with a greater likelihood of primary disease refractory to immune checkpoint inhibitors (ICIs) and worse overall survival. Strikingly, the association between antibiotics and survival rate was independent of tumor site, disease burden, and performance status, underscoring the direct detrimental effect of antibiotic-induced microbiota dysbiosis on cancer immunotherapy outcomes [261].

These findings collectively highlight the delicate interplay among antibiotic use, gut microbiota, and cancer therapy efficacy, emphasizing the need for careful consideration of antibiotic administration in oncological settings to minimize unintended consequences for treatment outcomes. Given these unwanted side effects, although effective in animal models, systemic treatment with broad-spectrum antibiotics may not be a proper strategy for targeting tumor-resident microbes in the real world [262].

The development of targeted antibiotics offers a promising alternative to eliminate certain detrimental bacteria while sparing others. In this context, Muñoz et al. [263] successfully identified lolamicin, a novel gram-negative-only antibiotic that targets the lipoprotein transport system specific to gram-negative pathogens over commensals with great efficacy both in vitro and in vivo. Despite this success, significant challenges remain in translating similar strategies into clinical practice. It is crucial to characterize the microbiota structures within tumors, the roles of specific taxa in cancer progression, and the characteristic differences between detrimental and beneficial microorganisms. In most cases, this task is difficult given the heterogeneity of intratumoral microbial communities and the complex interactions between these microbes and the TME. Moreover, the intracellular localization of intratumoral microorganisms poses an additional challenge. To reach these intracellular pathogens effectively, targeted antibiotics must be host-cell permeable. Therefore, a novel tumor-targeted and cell-permeable drug delivery system is required to ensure efficient antibiotic transmission to the desired sites.

Finally, both tumor-resident microbial communities and chemotherapeutic drugs significantly impact the efficacy of antibiotics, introducing additional challenges to integrating antibiotics into cancer treatment regimens. For example, in CRC, LaCourse et al. [264] demonstrated that 50% of patient-derived ex vivo CRC microbial communities, including *E. coli*, can metabolize 5-FU, a widely used chemotherapeutic agent and potent inhibitor of *Fusobacterium nucleatum*, into a nontoxic form. This transformation reduces the efficacy of 5-FU against both *Fusobacterium nucleatum* and cancer cells [264]. Additionally, Wang et al. [265] reported that chemotherapeutic drugs, such as etoposide, can induce the emergence of ciprofloxacin-resistant *Pseudomonas aeruginosa*. Consequently, bacterial biofilms that evolve in response to etoposide treatment were shown to protect cancer cells from the cytotoxic effects of the drug [265]. These studies highlight a complex interlinked network among intratumoral microbes, chemotherapeutic drugs, and antibiotics, which underscores the complexities and challenges associated with the use of antibiotics as part of cancer therapy.

### Nanotechnology and biomimetic approaches for targeted therapy

**Fig. 4:**
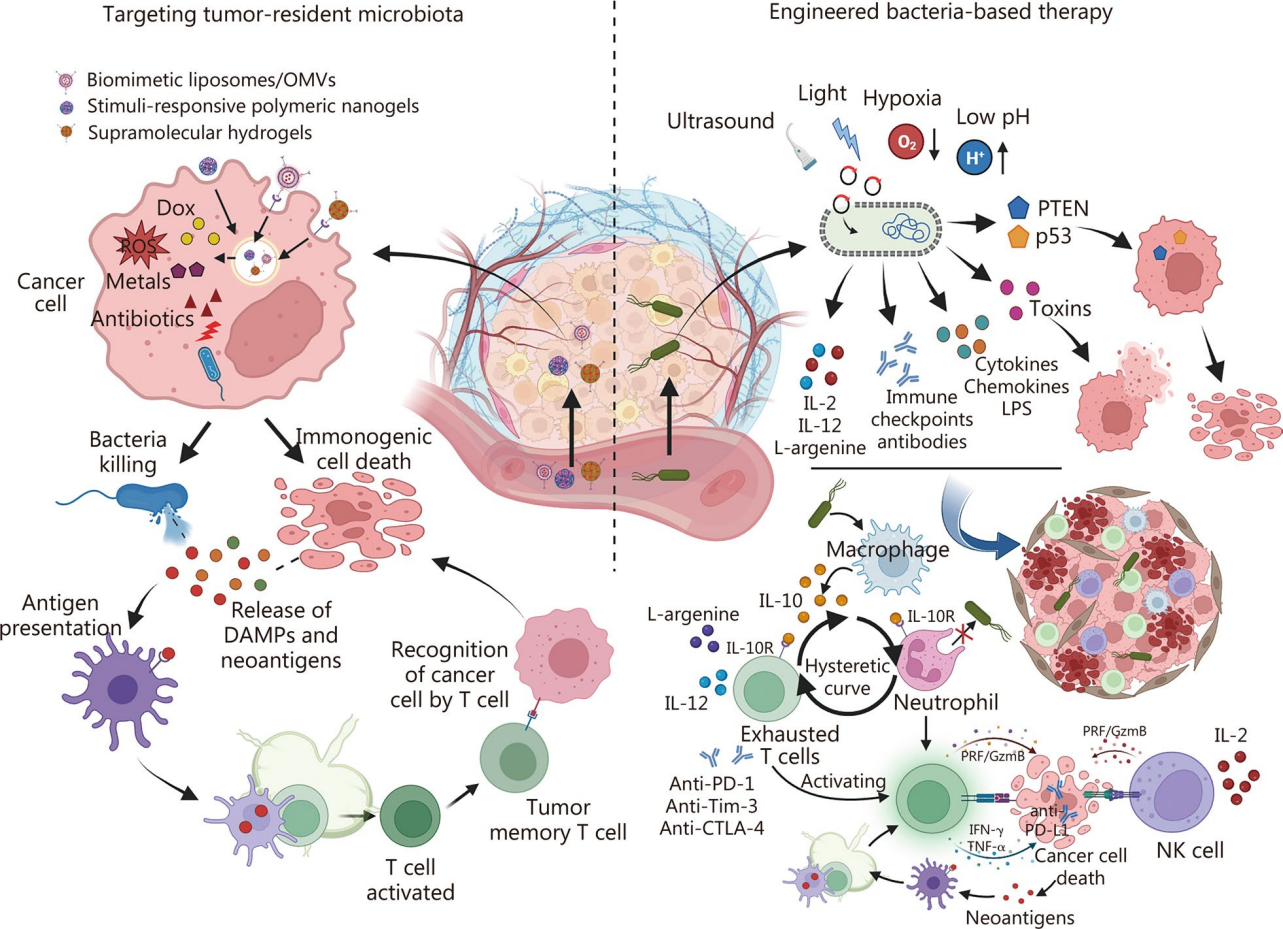
Emerging strategies for targeting tumor-resident microbiota and harnessing engineered bacteria for cancer therapy. On the left, nanoparticles, such as biomimetic liposomes/outer membrane vesicles (OMVs), stimuli-responsive polymeric nanogels, and supramolecular hydrogels, are designed to deliver both antibiotics and chemotherapeutics following their penetration into the tumor microenvironment, based on their ability to sense specific TME elements such as low pH and oxygen. Upon internalization into cancer cells, antibiotics and tumor-killing drugs are released, leading to efficient bacterial killing and enhanced immunogenic cell death. This, in turn, releases elevated levels of danger-associated molecular patterns (DAMPs) and neoantigens, which stimulate an efficient cancer-immunity cycle and activate tumor memory T cells that offer long-lasting protection against both infected and non-infected tumors. On the right, certain bacterial strains, such as *Listeria monocytogenes*, *Salmonella typhimurium*, and *Escherichia coli* MG1655, are engineered to penetrate the TME specifically and to express multiple therapeutic payloads, including cytotoxic molecules and immunomodulatory agents, under restricted control for their release. This results in the reprogramming of the tumor immune microenvironment (TIME) from a pro-tumor to an anti-tumor state. Cytokines such as IL-2 and IL-12 are released to activate T cells and NK cells. This is usually combined with the production of immune checkpoint antibodies to further eliminate immune suppression. Meanwhile, the engineered bacteria stimulate the production of IL-10, leading to a hysteretic response of neutrophils and T cells, allowing the bacteria to evade neutrophil phagocytosis and simultaneously stimulate anti-tumor T cell immunity. Dox doxorubicin, ROS reactive oxygen species, PTEN phosphatase, and tensin homolog deleted on chromosome ten, IL interleukin, LPS lipopolysaccharide, PRF perforin, GzmB granzyme B, IFN-γ interferon-γ, TNF-α tumor necrosis factor-α, PD-L1 programmed death-ligand 1. Created with BioRender.com

To address the limitations associated with antibiotic applications, recent research has focused on innovative methods that simultaneously target intracellular tumor-associated bacteria and cancer cells, utilizing advanced drug delivery systems and nanotechnology to increase the precision and efficacy of cancer treatments. Here, we summarize the emerging strategies for targeting tumor intracellular bacteria as shown in Fig. 4. The primary goal of this integrative strategy is to address bacterial infections and malignancies simultaneously, and for this purpose, both antibiotics and antitumor drugs have been integrated and effectively introduced into the TME. An example of such an approach is the metallosupramolecular nanogel, which is able to direct the release of antibiotics and chemotherapeutic agents, such as doxorubicin, from their configuration polymers at the site of the tumor, thus increasing the selectivity of the drug action and reducing adverse side effects [266]. Nanoparticles have also been engineered to sense specific TME characteristics, such as low pH and oxygen. Upon activation, nanoparticles release their payload, typically a combination of an antibiotic and a chemotherapeutic agent, directly into cancer cells. This design increases treatment efficacy while minimizing systemic side effects [267, 268].

Another notable advancement in targeting strategies is the development of biomimetic nanovehicles. These systems have demonstrated the potential to eradicate intracellular tumor-associated bacteria, a critical step in improving tumor immunotherapy. For example, inspired by the selective colonization of *Fusobacterium nucleatum* in cancer cells via the Fap2 lectin-Gal-GalNAc interaction, Liu et al. [269] engineered a nanoplatform. The aim of this novel platform, derived from the outer membrane vesicle (OMV) of *Fusobacterium nucleatum* and coated with iron and an antibiotic, metronidazole, is to obliterate intracellular *Fusobacterium nucleatum* and facilitate immunogenic cell death (ICD) in a triple-negative breast cancer model upon endocytosis. The killing of intratumoral bacteria leads to the release of pathogen-associated molecular patterns and activates tumor immunity, as dendritic cell maturation is subsequently accelerated to increase T-cell infiltration [269]. Similarly, Geng et al. [270] developed a biomimetic nanovehicle that incorporates membranes of *Fusobacterium nucleatum* and red blood cells loaded with doxorubicin and metronidazole. This strategy not only eliminated intratumoral *Fusobacterium nucleatum* but also synergized with PD-L1 blockade, thereby serving as an effective combination to treat cancer by employing both ICIs and direct targeting of the bacteria for a more effective response.

Indeed, the utilization of nanotechnology has emerged as a powerful platform to increase the efficacy of cancer therapy by addressing the shortcomings of current chemotherapy and radiotherapy, such as restricted intratumoral accumulation, limited immunogenicity, and systemic toxicity. In a recent review, Liu et al. [271] presented an extensive analysis to address the issues caused by an insufficient immune-stimulative effect of chemotherapy (ISECT), providing comprehensive insights into both mechanisms and strategies to mitigate an ISECT through nanotechnology, potentially by facilitating ICD, reversing the suppressive TIME, and strengthening the host’s systemic immune system to facilitate a successful cancer-immunity cycle [271, 272]. Representative nanomaterials designed for this purpose include thermal ablators (e.g., metal nanostructures), redox modulators (e.g., CaO₂), radiation sensitizers [e.g., gold (Au) nanoclusters], immune cell modulators [e.g., aluminum (Al)-containing nanomaterials], bacteria-based nanomaterials (e.g., engineered probiotic spores), and human cell membrane-based nanomaterials (e.g., cancer cell membrane-coated nanoparticles). These nanotechnologies have shown encouraging potential in mitigating an ISECT and initiating a robust cancer-immunity cycle in numerous preclinical studies [271].

Strikingly, the efficacy of nanotechnology in combating cancer could be significantly improved, particularly when it is combined with strategies that specifically target intratumoral bacteria. Interestingly, this synergy stems not only from the elimination of the detrimental effects of bacteria on cancer progression and therapy resistance but also from the unique capacity of tumor-resident bacteria to supply neoantigens that stimulate antitumor immunity. This capacity is supported by the observations that bacterial-specific T cells are cross-reactive with major histocompatibility complex class I (MHC-I) epitopes presented by cancer cells, thereby contributing to effective immune responses and offering alternative therapeutic opportunities, particularly for tumors with a low mutational burden and limited neoantigen availability [245, 273]. In a pivotal study using a CRC mouse model, Wang et al. [274] developed LipoAgTNZ, a pH-sensitive antibiotic liposome that encapsulates a silver-tinidazole complex, demonstrating great potential to eliminate intracellular *Fusobacterium nucleatum* within hypoxic orthotopic CRC tumors. This bacterial clearance exposes microbial epitopes homologous to tumor antigens, enabling their presentation via MHC-I and subsequent recognition by CD8⁺ T cells, which in turn establishes long-lasting antitumor immune memory capable of targeting both infected and uninfected cancer cells. Clinical observations further support this mechanism, as a retrospective analysis revealed that preoperative use of antibiotics targeting anaerobic bacteria was associated with significantly prolonged disease-free survival in patients with CRC, whereas postoperative antibiotic use conferred no such benefit [274]. This finding highlights a therapeutic window in which bacterial clearance can optimally prime antitumor immunity.

While these strategies show promise in preclinical studies, several challenges must be addressed before their clinical implementation. First, targeting mechanisms need to be optimized to increase specificity and efficacy while minimizing off-target effects. Second, the potential consequences of introducing these nanovehicles into the human body, including immunogenic responses and unintended impacts on the host microbiota, remain unclear. Third, the complexity of large-scale manufacturing, the lack of standardized toxicological evaluation frameworks, and other technical or regulatory hurdles further constrain the clinical translation of nanomedicines. Finally, increasing the availability of these novel technologies to patients in clinical settings necessitates extensive investigation in terms of consistent efficacy and safety in various patient groups. These issues must be resolved if these therapies are to progress from experimental to clinical status.

### Probiotics for the modulation of the cancer-associated microbiota

In addition to eliminating harmful microorganisms, the administration of probiotics may offer a safer way to reverse microbial dysbiosis to a healthy microbiota state, thus rendering probiotic administration a promising strategy for enhancing the efficacy of cancer therapy [275–278]. In light of this, the use of probiotics, particularly *Lactobacillus* spp. and *Bifidobacterium* spp., in CRC has been explored in several clinical trials where the intestinal mucosal-associated microbiota serves as the primary target [279–284]. Among these clinical trials, the most significant alteration observed was a decrease in the abundance of CRC-associated bacteria, such as Enterobacteriaceae, *Escherichia*, and *Fusobacterium* [280–282]. The decreases in the abundance of these bacteria may not only eliminate the detrimental role of these microorganisms but also inhibit their penetration into the tumor; however, validation of these results is warranted. Moreover, probiotics have been found to modulate immune cell responses and reduce inflammation, indicating a potential role of probiotics in regulating tumor immunity [281–284].

Emerging evidence underscores the potential of probiotics as adjunctive therapies to improve cancer treatment outcomes. For example, treatment with Colon Dophilus™, a probiotic formulation combining *Lactobacillus* and *Bifidobacterium* species, markedly reduces the incidence and severity of chemotherapy-induced diarrhea and enterocolitis [285], underscoring the therapeutic potential of probiotics in alleviating treatment-associated side effects. Furthermore, multiple probiotic taxa, including *Bifidobacterium*, *Lactobacillus*, and *Akkermansia muciniphila,* have been shown to increase the efficacy of ICIs and other immunotherapeutic strategies in preclinical models [286–288]. Mechanically, this effect is attributed to the modulatory role of probiotics in activating immune cells, such as dendritic cells and CD8^+^ T cells, thereby increasing their cytotoxic activity against cancer cells.

To date, many efforts have focused on the modulatory role of probiotics in the gut microbiome, but whether and how probiotics affect the intratumoral microbiota remains largely unknown. It is tempting to speculate that probiotics can inhibit the colonization of detrimental microorganisms in tumors by decreasing the number of cancer-associated microbes, reducing inflammation, and increasing epithelial integrity. Moreover, probiotics can accumulate within tumors and influence tumor dynamics. Shi et al. [286] demonstrated that *Bifidobacterium*, when systemically administered through gavage and intravenous injection, can accumulate within tumors and activate dendritic cells through STING signaling, indicating a direct role of probiotics in changing the TME. As such, it would be interesting to determine whether probiotics can directly modulate the intratumoral microbiota and how this interaction impacts TME and cancer progression.

Although the alteration of the intratumoral microbiota by native probiotics has rarely been explored, a recent study demonstrated that oral administration of engineered *Lactobacillus rhamnosus* GG probiotics functionalized with a gallium-polyphenol network (LGG@Ga-poly) led to a significant reduction in the abundance of tumor-promoting Proteobacteria and microbiota-derived lipopolysaccharides in pancreatic tumors [289]. Through this mechanism, LGG@Ga-poly markedly increased the efficacy of immune checkpoint blockade (ICB) therapy [289]. This study underscores the potential of probiotics to combat tumors by modulating the intratumoral microbiota, offering a promising avenue for improving cancer immunotherapy.

Despite growing interest in probiotics as supplementary cancer treatments, unexpected findings highlight the need for careful evaluation of their clinical applications. For example, in a large cohort study of patients with melanoma (*n* = 438), the authors reported that patients who consumed commercially available probiotics, primarily *Bifidobacterium longum*- or *Lactobacillus rhamnosus* GG-based probiotics, were prone to experience reduced progression-free survival (PFS) and decreased odds of response to ICB [290]. Notably, patients who consumed sufficient dietary fiber but no probiotics presented significantly longer PFS and higher ICB response rates than those who consumed both dietary fiber and probiotics [290]. These observations indicate that commercially available probiotics may negatively influence the outcomes of cancer immunotherapy. Using multiple mouse models, the authors demonstrated that the use of probiotic was associated with a poorer response to anti-PD-1 treatment [290]. This poor anti-PD-1 treatment response was accompanied by significantly reduced gut microbiome diversity and a decrease in the number of IFN-γ^+^CD8^+^ T cells within tumors, suggesting a compromised antitumor immune response [290]. This finding contradicts another study on the use of probiotics in cancer treatment [279], with the discrepancy potentially stemming from differences in the probiotic strains administered, cancer types, patient microbiome compositions, and study designs-factors that warrant further investigation. This study highlights the potential “dark side” of probiotics, emphasizing the need for a comprehensive understanding of their effects on tumor dynamics and cancer therapy before their clinical implementation.

### Engineered bacterial systems for precision therapy

The selective colonization of certain bacteria in cancer cells has inspired research into the development of engineered bacterial platforms as drug-delivery vehicles. In this strategy, the intrinsic ability of bacteria to penetrate deep into the TME, where traditional therapies often fail to reach, is exploited. Advances in synthetic biology have further enabled precise control over bacterial behavior, allowing for restricted and conditional release of antitumor payloads, such as immunomodulatory agents, cytotoxic molecules, and tumor-specific neoantigens, to initiate and sustain the cancer-immunity cycle. Moreover, the inherent capacity of bacteria to modulate immune responses renders them ideal mediators for bolstering both innate and adaptive antitumor immunity. These engineered systems not only facilitate spatially confined immune activation but also increase therapeutic efficacy and safety through spatiotemporal control [291]. These strategies are summarized in Fig. 4.

For example, engineered *E. coli* MG1655 cells coated with lanthanide upconversion nanoparticles (UCNPs) were observed to accumulate at hypoxic tumor sites due to their innate chemotactic behavior [292]. This accumulation facilitated localized near-infrared (NIR) laser irradiation, which triggered the conversion of NIR light to blue light by the UCNPs, subsequently activating the bacteria to secrete HlyE perforin and effectively kill cancer cells [292]. In this way, tumors can be specifically targeted with limited side effects on healthy tissues. Another example involves engineered attenuated *Salmonella* strains carrying genes for PD-1 and Tim-3 single-chain variable fragment antibodies (scFvs) [293]. These bacteria were found to colonize the hypoxic tumor core and produce PD-1 and Tim-3 scFvs to target exhausted PD-1^+^Tim-3^+^ T cells. As a consequence, exhausted T cells were revived, leading to the secretion of IFN-γ and an enhanced capacity to kill cancer cells [293]. Moreover, engineered bacteria elicited a strong innate immune response and further activated T cells, underscoring the great value of engineered bacteria in reshaping the TIME and increasing the efficacy of immunotherapy [293]. In addition, genetically modified bacteria such as *Listeria monocytogenes* and *Salmonella typhimurium* could express tumor-associated antigens, facilitating targeted delivery and localized expression, which could increase the antitumor immune response [294]. Furthermore, these engineered bacteria have been employed as carriers for gene therapy and immunotherapy, demonstrating the capability to directly disrupt tumor metabolism and signaling pathways [295].

Encouraged by the potency of bacterial immunotherapy in preclinical studies, clinical trials have been conducted to test the efficacy and tolerance of these bacteria for the treatment of multiple malignancies [291, 296]. For example, in a phase, I dose-escalation (10^5^ – 10^10^) trial, single-dose oral administration of attenuated *Salmonella typhimurium* encoding human IL-2 (Saltikva) was found to be safe in patients with metastatic gastrointestinal cancers [297]. Although no survival advantage was observed, treatment with Saltikva increased the circulating NK and natural killer T (NKT) cell populations, and further multiple-dose studies are warranted [297]. Despite these developments, the clinical translation of bacterial immunotherapy remains a considerable challenge. Numerous clinical trials have been terminated due to limited efficacy or safety issues. To date, Bacillus Calmette-Guérin, a live attenuated *Mycobacterium tuberculosis* vaccine, remains the only FDA-approved bacterial agent for the treatment of high-risk and nonmuscle-invasive bladder cancer [298]. Several key challenges must be addressed, including safety and off-target risks, limited tumor-targeting specificity, and stringent regulatory, manufacturing, and quality control requirements [291, 296].

To address these challenges, a deeper mechanistic understanding of how tumor-targeting bacteria modulate the TIME is essential. A recent study by Chang et al. [299] provided important mechanistic insights into how tumor-targeting bacteria evade immune surveillance and, paradoxically, stimulate an antitumor response at the same time. In this study, an attenuated *Salmonella typhimurium* strain was engineered [designer bacterium 1 (DB1)] with genetic circuits designed to induce bacterial lysis under aerobic conditions (*asd* under the control of *P*_*hypo*_) and simultaneously increase tumor penetration (*hly* under the control of *P*_*sseA*_), thereby enabling tumor-specific bacterial colonization with increased safety and functional precision [299]. Using this platform, the authors demonstrated that intratumoral DB1 activated TLR4 signaling in tumor-associated macrophages (TAMs), leading to IL-10 production and subsequent upregulation of IL-10 receptor (IL-10R) expression on multiple immune subsets, including CD8⁺ T cells, TAMs, and tumor-associated neutrophils (TANs) [299]. Therefore, a hysteresis loop is established in which sustained IL-10R expression allows these immune cells to remain responsive to IL-10 even after cytokine levels decline. As a result, the phagocytic activity of TANs against DB1 was suppressed, while exhausted tumor-resident memory-like CD8⁺ T cells were reactivated, ultimately achieving durable antitumor protection [299].

This study expands our understanding of the potential crosstalk between bacteria, either engineered to target tumors or native tumor-resident microbes potentially involved in cancer progression and the TIME to allow sustained intratumoral bacterial survival and immune reprogramming. For engineered bacterial systems, leveraging this cytokine hysteretic response of tumor-infiltrating immune cells may provide a strategy to potentiate the antitumor immune response stimulated by tumor-targeting bacteria, which could be further strengthened by incorporating a drug delivery system and nanotechnology. For native tumor-resident microbes, although further investigation is needed, a similar mechanism may also contribute to their selective colonization, immune evasion, and ability to reshape the TIME landscape. For example, in the study by Galeano et al. [109], infection with *Fusobacterium nucleatum* led to the recruitment of neutrophils into CRC spheroids and induced the formation of neutrophil clusters, a phenomenon also observed within bacteria-colonized niches in clinical tumor samples. Therefore, further investigation into whether a similar mechanism enables tumor-resident bacteria to evade neutrophil phagocytosis while simultaneously reprogramming the TIME is warranted. If this is the case, several critical questions arise, such as whether this mechanism is applicable across diverse bacterial taxa and tumor types or specific to certain host-microbe interactions and how it contributes to microbiota-driven immune suppression within tumors. Such insights may help in the development of novel therapeutic strategies to reverse microbiota-induced immunosuppression within tumors, prevent or limit the colonization of immunosuppressive intratumoral microbes, and harness engineered bacterial platforms to strengthen antitumor immunity in a spatially controlled and immunologically favorable manner.

With the advancement of bacterial delivery systems, concerns have emerged regarding the potential side effects of unintended bacterial infections. To address this issue, engineered probiotics are considered as a superior platform for implementing this technique, with multiple studies that have been conducted achieving great efficacy. For example, in a recent breakthrough, the probiotic strain *E. coli* Nissle 1917 was bioengineered to increase L-arginine concentrations, promoting T-cell infiltration into TME [300]. This modality, when synergized with PD-L1 inhibitory antibodies, strengthened antitumoral responses [300]. A recent study revealed the colonization of engineered *E. coli* Nissle 1917 carrying the oncogene phosphatase and tensin homolog in CRC tumors, which inhibited the growth of cancer cells in a mouse model [301]. Similarly, Liao et al. [302] designed and developed an engineered probiotic based on *E. coli* Nissle 1917. This engineered bacterium specifically targets and colonizes tumors and continuously releases anti-PD-1 and IL-12 to increase the response of tumors to ICB [302]. These studies highlight the capacity of probiotic platforms in targeted cancer therapy. Considering the potential of probiotics to modulate the intratumoral microbiota and local immunity, further studies are warranted to leverage these microorganisms to develop multifunctional therapeutic strategies.

In addition to the use of probiotics as alternative drug delivery systems, advancements in nanotechnology have enabled the creation of bacteria-like nanorobots that increase the precision of drug targeting and drug efficacy. In this context, artificial bacterial OMVs can transport chemotherapeutic agents and modulators directly into the tumor, achieving local and effective therapeutic action with a lower overall incidence of side effects [303, 304]. In addition, nonviral polymer systems have been developed to mimic the geometric and surface characteristics of viruses, which effectively deliver therapeutic genes into cancer cells, showing potential in overcoming tumor resistance [305]. These techniques make it possible to overcome the limitations of using live organisms while retaining the advantages of precise targeting and effective therapeutic delivery.

In conclusion, the selective colonization of certain microorganisms in tumors and their roles in tumor dynamics underscores both the need to target them and their potential utility as drug delivery vehicles. Although emerging strategies are promising for fighting against detrimental tumor-resident microbes, several challenges remain to be addressed. These challenges include the heterogeneity of the microbiota across individuals, which can affect therapeutic outcomes, and the complexity of manipulating these communities without disrupting beneficial microbes. Moreover, significant hurdles are encountered in clinical translation, including ensuring safety and efficacy across diverse patient demographics. For the development of drug delivery systems leveraging these microorganisms, concerns about potential infections or unintended immune reactions also highlight the critical need for careful consideration in engineering and clinical applications.

## Discussion and conclusion

As our understanding of the intricate relationship between tumor-resident microbiota and cancer deepens, the potential to harness these microbial communities for therapeutic applications becomes increasingly apparent. Intracellular microbiota, in particular, challenges traditional boundaries between microbial infection and cancer biology. These microbes contribute to genetic and epigenetic modifications and modulate host immune responses, establishing themselves as critical mediators of tumor behavior, influencing processes from initiation and progression to metastasis and therapy response.

Despite significant advancements, substantial gaps remain in understanding the roles of intracellular bacteria compared to those in the extracellular TME. Key questions include how bacteria selectively colonize cancer cells, persist within hostile cellular environments, and influence tumor dynamics. Current research often focuses on associations between bacteria and tumor characteristics, with limited exploration of causation and underlying mechanisms, leaving the full extent of their impact on cancer evolution unclear. Additionally, the heterogeneity of microbial species across cancers and individuals underscores the complexity of these interactions, which is often overlooked by research methodologies that fail to fully capture the variability within the TME and the systemic effects of microbiota on host physiology.

Addressing these challenges will require advanced genomic, proteomic, and bioinformatics tools to map interactions between intracellular microbiota and cancer cells comprehensively. Interdisciplinary approaches will be useful in targeting the mapping of interactions of intracellular microbiota. It is also advisable for future work to investigate the exact intracellular events caused by the bacterial interaction with the cancer cell and to find out whether any of the events can be utilized for therapy. Besides, new models that depict much closer similarities to the human tumor milieu need to be developed to achieve a successful transfer of the knowledge obtained in the laboratory to clinical practice.

In summary, the cancer-associated microbiome, especially the ones residing and proliferating within the cancer cells, are potential therapeutic targets that are yet to be fully understood. There is an urgent need for closer collaboration among microbial, cancer, and synthetic biologists in order to come up with precise and personalized microbiota-driven treatment approaches in managing cancer patients. The combination of microbiota-targeted cancer therapy with other cancer therapeutic strategies, including chemotherapy, radiotherapy, and immunotherapy may provide powerful synergistic effects, enhancing therapeutic efficacy and potentially overcoming resistance mechanisms. By targeting the unique interactions between cancer cells and the intratumoral microbiome, such combination therapies could reprogram the TME and bolster immune responses, holding the promise of redefining therapeutic approaches and improving prognostic outcomes for patients with various cancers.

## Data Availability

Not applicable.

## Abbreviations

5-FU: 5-Fluorouracil
AUC: Area under the curve
CAR: Chimeric antigen receptor
CCL: C–C motif chemokine ligand
CRC: Colorectal cancer
CXCL: C-X-C motif chemokine ligand
DB1: Designer bacterium 1
DNA: Deoxyribonucleic acid
EAC: Esophageal adenocarcinoma
ECM: Extracellular matrix
ER: Endoplasmic reticulum
FAK: Focal adhesion kinase
Gal-GalNAc: D-galactose-β(1–3)-N-acetyl-D-galactosamine
HCC: Hepatocellular carcinoma
hnRNP: Heterogeneous nuclear ribonucleoprotein
HPV: Human papillomavirus
*H. pylori*: *Helicobacter pylori*
ICB: Immune checkpoint blockade
ICD: Immunogenic cell deathICIs: immune checkpoint inhibitors
IL: Interleukin
IL-10R: IL-10 receptor
ISECT: Insufficient immune-stimulative effect of chemotherapy
LTS: Long-term survival
MDSCs: Myeloid-derived suppressor cells
MHC-I: Major histocompatibility complex class I
MMP: Matrix metalloproteinase
MSI: Microsatellite instability
NK: Natural killer
NIR: Near-infrared
OMV: Outer membrane vesicle
OSCC: Oral squamous cell carcinoma
OUT: Operational taxonomic unit
OV: Ovarian cancer
PDAC: Pancreatic ductal adenocarcinoma
PD-L1: Programmed death-ligand 1
PFS: Progression-free survival
ROC: Receiver operating characteristic
SCFAs: Short-chain fatty acids
scFvs: Single-chain variable fragment antibodies
scRNA-seq: Single-cell RNA sequencing
TAMs: Tumor-associated macrophages
TANs: Tumor-associated neutrophils
TFEB: Transcription factor EB
TJs: Tight junctions
TIME: Tumor immune microenvironment
TLR4: Toll-like receptor 4
TME: Tumor microenvironment
TNF: Tumor necrosis factor
UCNPs: Upconversion nanoparticles
UPEC: Uropathogenic *E. coli*
WT: Wild-type
